# Biliverdin reductase: a target for cancer therapy?

**DOI:** 10.3389/fphar.2015.00119

**Published:** 2015-06-03

**Authors:** Peter E. M. Gibbs, Tihomir Miralem, Mahin D. Maines

**Affiliations:** Department of Biochemistry and Biophysics, University of Rochester School of Medicine and Dentistry, Rochester, NY, USA

**Keywords:** biliverdin reductase, protein kinase C, extracellular signal-regulated MAP kinases, heme oxygenase-1, oxidative stress, cell cycle

## Abstract

Biliverdin reductase (BVR) is a multifunctional protein that is the primary source of the potent antioxidant, bilirubin. BVR regulates activities/functions in the insulin/IGF-1/IRK/PI3K/MAPK pathways. Activation of certain kinases in these pathways is/are hallmark(s) of cancerous cells. The protein is a scaffold/bridge and intracellular transporter of kinases that regulate growth and proliferation of cells, including PKCs, ERK and Akt, and their targets including NF-κB, Elk1, HO-1, and iNOS. The scaffold and transport functions enable activated BVR to relocate from the cytosol to the nucleus or to the plasma membrane, depending on the activating stimulus. This enables the reductase to function in diverse signaling pathways. And, its expression at the transcript and protein levels are increased in human tumors and the infiltrating T-cells, monocytes and circulating lymphocytes, as well as the circulating and infiltrating macrophages. These functions suggest that the cytoprotective role of BVR may be permissive for cancer/tumor growth. In this review, we summarize the recent developments that define the pro-growth activities of BVR, particularly with respect to its input into the MAPK signaling pathway and present evidence that BVR-based peptides inhibit activation of protein kinases, including MEK, PKCδ, and ERK as well as downstream targets including Elk1 and iNOS, and thus offers a credible novel approach to reduce cancer cell proliferation.

## What is BVR?

Some 50 years ago, an enzymatic activity that reduced biliverdin to bilirubin was described (Singleton and Laster, 1965; Tenhunen et al., 1970) and subsequently named biliverdin reductase (BVR). Some years later, highly purified preparations of the rat enzyme revealed a unique profile for the reductase; at near-neutral pH, the enzyme preferentially uses NADH, while under more alkaline conditions (pH ∼8.4) NADPH is the preferred cofactor (Kutty and Maines, 1981). Subsequent studies with the human and bovine enzymes indicated that the dual pH/cofactor activity profile is preserved in other mammalian species (Rigney and Mantle, 1988; George et al., 1989; Ding and Xu, 1994), and a functional reductase has been isolated from cyanobacteria (Schluchter and Glazer, 1997). Advances in genomic sequencing have revealed the presence of genes encoding the reductase in a wide variety of vertebrates, including fish, amphibia, reptiles and birds.

Attempts at further characterization of the dual pH/cofactor profile of the enzyme led to a series of findings indicating a much wider role for the enzyme than had been expected. The protein could be resolved by isoelectric focusing into multiple variants that were found to arise from phosphorylation (Huang et al., 1989a,b,c; Huang and Maines, 1990). This in turn led to the observation that BVR is itself a protein kinase (Salim et al., 2001; Lerner-Marmarosh et al., 2005), and that it belongs to the relatively rare class of dual specificity kinases that phosphorylate tyrosine in addition to serine and threonine.

The primary structure of human BVR harbors an unparalleled number of functional sequence motifs that enable BVR to act as a regulator of signal transduction pathways. It contains a bZip DNA binding sequence, and canonical nuclear localization sequence (NLS) and nuclear export sequence (NES) that together facilitate bidirectional nuclear transport of BVR (Lerner-Marmarosh et al., 2008) and enable it to function as a transcription factor (Ahmad et al., 2002). There are also sequence motifs that are characteristic of signal transduction proteins (Gibbs et al., 2012b). BVR has two consensus *Src*-homology 2 (SH2) -binding motifs that resemble binding sites in substrates of the insulin receptor kinase (IRK); the tyrosine residues in these motifs, Y^198^ and Y^228^ in the YMKM and YLSF motifs, respectively, are targets of IRK (Lerner-Marmarosh et al., 2005). The SH2 domains were also predicted to interact with receptors, including the Toll-like membrane glycoproteins, via tyrosine-phosphorylated motifs in the latter (Wegiel et al., 2011). In addition, the observed separation of kinase and regulatory domains resembles, to some extent, the organization of the protein kinase C (PKC) isozymes (Newton, 1995). The dinucleotide/ATP-binding site of BVR (Rossmann fold) is located in the catalytic domain in the N-terminal region of the molecule (Whitby et al., 2002). A prominent feature of the C-terminus is a large six-stranded β-sheet that is a candidate domain for protein: protein interactions. Additional regulatory sequences are concentrated in an extended C-terminal α-helix. The regulatory domain includes two consensus binding sites, the C- and D-Box, that respectively display high- and low-affinity for kinases in the mitogen-activated protein kinase (MAPK) pathways (Myers et al., 1996; Minden and Karin, 1997; Jacobs et al., 1999). The D-Box of human BVR also overlaps with a Zn(II) binding cysteine-rich sequence that resembles similar motifs found in the C2 regulatory domains of PKCs (Steinberg, 2008; Newton, 2010). This cysteine-rich sequence also has the potential to respond to the redox status of the cell, and could function as a signaling switch, for example in oxidative stress. Each of these motifs has been implicated in aspects of BVR signaling (Kapitulnik and Maines, 2009; Gibbs et al., 2012b), although each signaling event depends on a discrete subset of motifs, as would be expected from the extensive array of pathways where BVR functions. It has become apparent that these motifs are used by BVR to mediate activities of regulatory protein kinases, although not all kinases are activated by the same mechanism. Thus BVR can function as a dual-specificity kinase, as a scaffold independent of kinase function, and also as a transport protein to translocate other signaling molecules.

**FIGURE 1:**
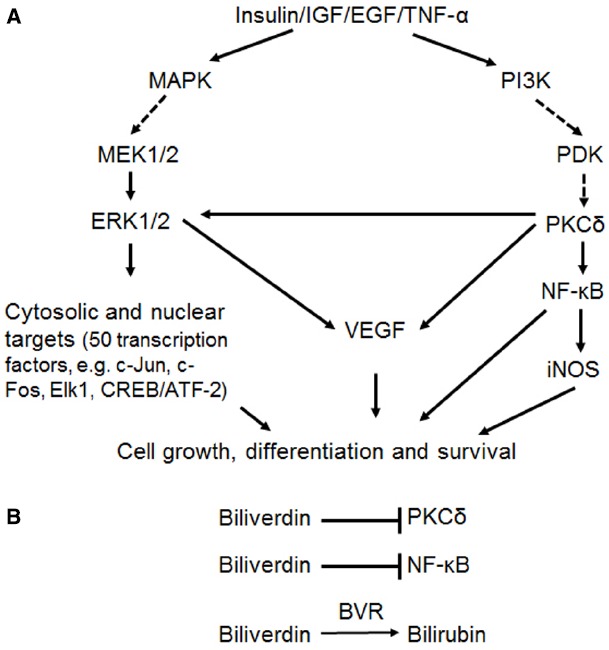
**(A)** Simplified representation of the two major arms of signal transduction from cellular receptors, in particular, those for insulin and growth factors. Solid arrows indicate reactions wherein BVR has been demonstrated to act as a scaffold, molecular bridge, intracellular transporter, transcription factor or kinase. In the case of activation of VEGF expression by ERK1/2 and/or PKCδ, this follows as a consequence of BVR-dependent stimulation of kinase activities. **(B)** Three mechanisms of BVR function as a cytoprotectant. The reductase activity converts biliverdin to the antioxidant bilirubin. In addition, biliverdin is a known inhibitor of PKCδ (Miralem et al., 2012) and of NF-κB (Gibbs and Maines, 2007).

The range of cell signaling functions mediated by BVR is illustrated in Figure 1. In response to extracellular stimuli, such as hormones, growth factors or cytokines, two major signaling pathways are activated, the MAPK and phosphatidylinositide 3-kinase (PI3K) pathways. As will be discussed, BVR activates kinases in both pathways, with the end result being expression of genes that are involved in cell growth, differentiation and survival. This, in turn, suggests that BVR is of direct relevance in cancer development, where unimpaired cell proliferation is the common outcome.

Each of the extracellular stimuli acts by binding to a cell surface receptor, resulting in transduction of a signal through the cell membrane. In the case of insulin and insulin-like growth factor-1 (IGF-1), this activates a kinase domain located on the cytoplasmic surface of the membrane, triggering cascades of downstream signals. It was demonstrated that IRK can use BVR as a substrate (Lerner-Marmarosh et al., 2005), and in turn BVR is able to phosphorylate insulin receptor substrate-1 (IRS-1), a major IRK substrate that acts to limit glucose uptake in response to insulin. Overexpression of BVR itself results in diminished glucose uptake, presumably mediated by the phosphorylation of IRS-1, whereas increased uptake is observed after treatment with small interfering RNA (siRNA) to attenuate cellular BVR levels (Lerner-Marmarosh et al., 2005).

Here we discuss how BVR and its signaling is integrated into carcinogenesis and cancer progression, and how peptides, based on well characterized motifs in human BVR, most likely will be of use in cancer prevention or suppression. Two major pathways that regulate cell proliferation survival and invasion, i.e., Ras-MAPK and PI3K, play central roles in multiple processes in cancer (Malumbres and Barbacid, 2003). Both cascades have input from BVR at multiple steps, and the modes of activation by BVR will be discussed.

## BVR in Cancer

Cancer is a heterogeneous group of disorders that are characterized chiefly by unconstrained cell replication, and by invasiveness of the cancer cells that eventually enables them to metastasize throughout the organism. Cancers arise as the end result of accumulated genetic and epigenetic adaptations that enable cells to escape the homeostatic controls that ordinarily suppress cell proliferation while inhibiting survival of aberrantly proliferating cells, although the breakdown in control is not identical in all cancers (Evan and Vousden, 2001). These adaptations may occur in any of a variety of signaling pathways, such that the etiology of two given tumors may be completely different. In addition to these changes, progression of the cancer is associated with a complex interplay among tumor cells, the surrounding non-neoplastic cells and the extracellular matrix (Leber and Efferth, 2009). The tumor cells develop several novel features, including hyperproliferation, resistance to apoptosis, metabolic changes, genomic instability, induction of angiogenesis and increased migration (Leber and Efferth, 2009). Most of these characteristics are regulated by cellular signal transduction. Aberrant signaling in pathways that are intimately involved in embryogenesis and adult tissue homeostasis, such as those regulated by the extracellular ligands Wnt and Hedgehog, or by the cell surface receptor Notch, have been definitively associated with initiation and progression of tumorigenesis (Jiang and Hui, 2008; Klaus and Birchmeier, 2008; Sethi and Kang, 2011). The cytokine tumor-derived growth factor-β exerts paradoxical effects on cells—it acts as a tumor suppressor, such that transformation necessitates evasion of the cytokine, but at the same time may mediate changes in the microenvironment of the cell to facilitate invasiveness and in suppression of the immune system, both of which would be expected to promote tumor growth (Massague, 2008; Inman, 2011). It is beyond the scope of this review to discuss these pathways in detail and currently the relevance of BVR in any of the pathways is tenuous at best. Rather, we will focus on the pathways that converge on the extracellular-signal-regulated kinases 1 and 2 (ERK1/2), for which a considerable body of evidence has accumulated indicating that BVR is a regulator of their activation. Other points of discussion will include the role of BVR in the PI3K/Akt pathway, and its effects on NF-κB (nuclear factor κ-light-chain-enhancer of activated B cells), both of which are relevant to the etiology and maintenance of cancer cells. It should also be noted, however, that there is considerable cross-talk among all of the pathways, such that Akt functions in Wnt signaling, although there is evidence to suggest that the mechanism differs between normal and tumor cells (Anderson and Wong, 2010), and the smad2 and smad3 proteins in the TGF-β pathway are activated by ERK (Hough et al., 2012). The relevance of BVR may not, therefore, be entirely limited in scope.

**FIGURE 2:**
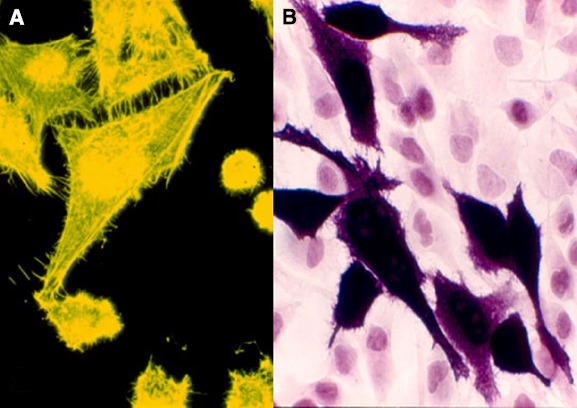
**Morphology of cultured cells with overexpressed BVR. (A)** HEK cell transfected with an EGFP-BVR fusion plasmid shows a flattened morphology atypical of the parent cell line. Adapted from (Maines, 2005). **(B)** Similarly, HeLa cells transfected at low efficiency with a human BVR expression plasmid and analyzed by immunohistochemistry with antibody against BVR are apparently enlarged relative the surrounding lawn of untransfected cells.

**FIGURE 3:**
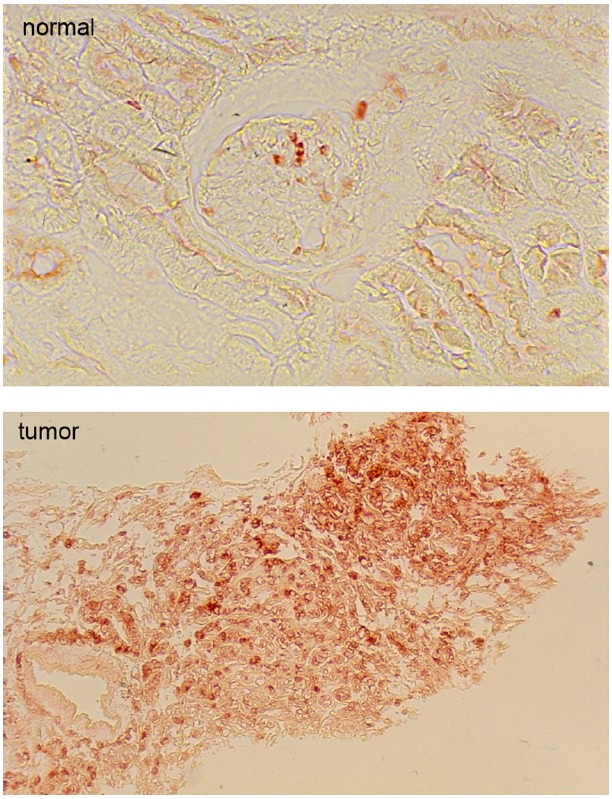
**Immunohistochemical detection of biliverdin reductase in normal human kidney cells and in a kidney tumor.** Note that the magnification of the normal kidney is 10-fold greater than that of the tumor. Modified from (Maines et al., 1999).

As shown in Figure 2, overexpression of BVR leads to changes in cell morphology. In Figure 2A, the transfected cells appear to be flattened, and more spread out on the plate. This is also evident in Figure 2B, where transfected cells overexpressing BVR are seen on a background of untransfected cells, which display a more rounded morphology. Moreover, it was observed that in renal tumors, BVR was both overexpressed and located predominantly in the nucleus (Figure 3; Maines et al., 1999). Elevated BVR expression was not limited to the tumor cells as such, but was also seen in infiltrating T-cells, monocytes, macrophages and lymphocytes. Increased BVR was also seen in circulating lymphocytes from the same patient. It was also noteworthy that reductase activity in tissue extracts was increased in the tumors; however, this was dependent on the assay. The activity was increased twofold if NADH (at pH 6.7) was used as cofactor, while there was minimal change with NADPH at pH 8.4. These observations offer a potential explanation of the elevated levels of heme oxygenase-1 (HO-1) seen in prostate cancers as first noted by (Maines and Abrahamsson, 1996). In this early study, HO-1 in normal prostate tissue is expressed in the stroma, and the epithelial layers of acini and ducts. Benign prostate hyperplasia show elevated HO-1, particularly in the basal layers of epithelia, whereas in undifferentiated prostate cancer, all cells, including those of blood vessels, show high HO-1. The constitutively expressed HO-2 showed no change in expression. These observations have been confirmed and extended, further demonstrating that HO-1 localizes in the nuclei of prostate tumor cells, and counters, in part at least, the activation and/or expression of proteins involved in neovascularization (Sacca et al., 2007; Ferrando et al., 2011, 2013; Biswas et al., 2014). It is tempting to speculate that the elevated expression of HO-1 could be a consequence of elevated BVR levels, since BVR has been shown to play a role in HO-1 induction (Ahmad et al., 2002; Kravets et al., 2004; Miralem et al., 2005; Tudor et al., 2008). However, some prostate cancers have recently been shown to have elevated levels of flavin oxidase (Pallua et al., 2013). This enzyme (also known as biliverdin reductase B, BVR-B) is unrelated to BVR (as used in the context of this review), but is the primary source of bilirubin in the fetus. As such, the increased expression could reflect the tumor cells being at a very primitive stage of differentiation. Alternatively, it could reflect the hypoxic state of the tumor; BVR-B was over-expressed in skeletal muscle of members of an expedition to Mt Everest after 19 days of the low O_2_ pressure characteristic of 5300 m above sea level (Levett et al., 2014). Other than being a possible source of bilirubin, the relevance of this enzyme to cytoprotection in cancer and the functions of BVR in general, is unclear.

The possibility that BVR expression levels could be used as a biomarker of certain cancers (Hellman et al., 2009) was suggested by proteomic approaches that compared proteins in biopsies obtained from normal vaginal epithelium, primary vaginal carcinomas and primary cervical carcinomas. Significantly altered expression of 43 proteins was observed between the tumors and non-tumor tissue, with 26 of these being up-regulated and the remaining 17 down-regulated. Expression of three proteins, erbB3-binding protein, the helicase DDX48 and BVR was found to be altered solely in vaginal carcinomas and therefore were proposed as useful molecular markers for this carcinoma. The elevated levels of BVR in tumor cells is predictably due to a cancer-related hypoxia, as it was shown that BVR gene expression can be induced in hypoxic HEK293 cells or in cells over-expressing the HIF1 transcription factor (Gibbs et al., 2010). Similarly,(Song et al., 2013) have shown that in pulmonary arterial smooth muscle cell (PASMC) apoptosis is inhibited by hypoxia, and BVR protein and mRNA levels were upregulated. Treatment of PASMC with siRNA against BVR led to increases in markers of apoptosis (Song et al., 2013). Furthermore, these effects could be reversed with bilirubin, which could itself block apoptosis if the ERK1/2 pathway was active. It is noted that hypoxia induces alterations in the cellular redox status, which in turn plays a critical role in the subsequent development of resistance to chemotherapeutic agents in cancer cells. BVR expression in human glioblastoma cells was significantly increased in response to hypoxia (Kim et al., 2013). In contrast, the hypoxia-induced chemoresistance in these cells could be attenuated by treatment with siRNA against BVR, which led to significantly increased levels of intracellular reactive oxygen species (ROS). Pretreatment of the cells with the antioxidant *N*-acetylcysteine reversed the sensitivity to chemotherapeutics seen in BVR-depleted cells. Enhanced activity of BVR also protected cells from treatment with genotoxic anti-cancer drugs such as cisplatin or doxorubicin (Florczyk et al., 2011). This effect predictably could be linked to activation of PKCs α and β, as BVR inhibition reversed the protection (Florczyk et al., 2011). The activity of BVR thus would influence the effectiveness of therapies. It is noted that this is a narrow range of studies, and that while there is a correlation of overexpression of BVR and tumor growth, one cannot distinguish whether the elevated BVR levels are a cause or an effect of the cellular reprogramming that occurs in cancer. That may not however, disqualify BVR as potential target for therapy.

**FIGURE 4:**
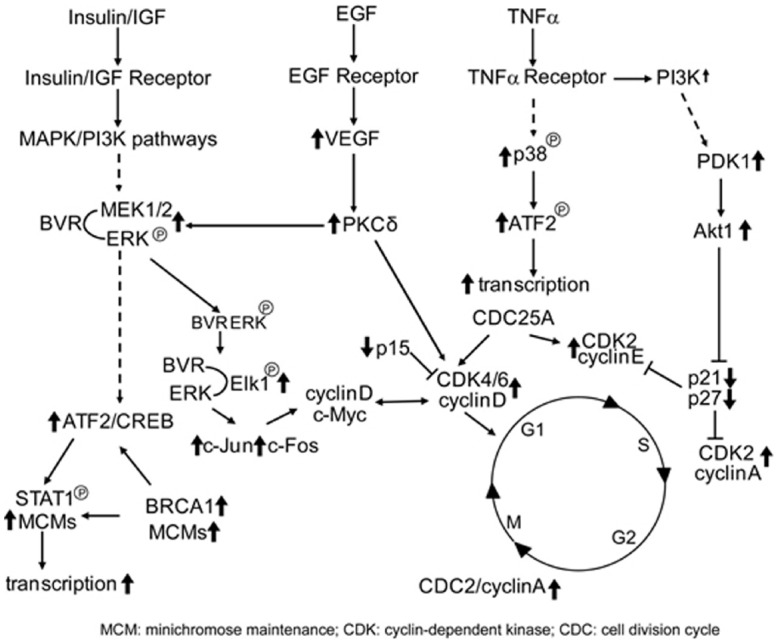
**BVR targets in cell cycle activation.** Signaling pathways that directly activate the cell cycle and themselves activated by BVR are shown. Those proteins that are activated by BVR or whose levels are modulated by overexpression of BVR (such as the cyclins, CDKs, and CDK inhibitors, as described in Table 1) are indicated by bold arrows.

Given the nuclear localization of BVR seen in kidney tumors (Maines et al., 1999), and its ability to activate transcription factors linked to cell growth, this result is a tempting indication of BVR perhaps being directly involved in tumor cell growth. In this respect, it is worth noting that overproduction of BVR results in significant changes in expression of cell cycle genes (Table 1), including transcription factors, genes involved in chromosome replication and cyclin-dependent kinases, while expression of two check-point inhibitor protein genes are significantly down regulated (Kravets et al., 2004). These findings indicate a role for BVR regulation of the cell cycle. This either occurs directly due the pronounced relocation of BVR to the nucleus (Figure 3), or indirectly by activation of upstream pathways that themselves activate the cell cycle (Figure 4).

**TABLE 1:** **Cell proliferation genes activated by over-expressed BVR**.

**Gene name**	**Fold-change**
Cullin-1 (CUL-1)	107
Mini-chromosome maintenance-3 (MCM3)	21.3
Cell division cycle 25 homolog A (CDC25A)	15.8
E2F transcription factor-3 (E2F-3)	13.5
Ring-box protein-1 RBX1)	8.6
Proliferating cell nuclear antigen (PCNA)	7.0
Cyclin-dependent kinase-2 (CDK2)	4.9
Cyclin-dependent kinase-4 (CDK4)	4.0
CDK inhibitor 2B (CDKN2B) p15	–2.5
CDK inhibitor 1B (CDKN1B) p27^KIP^	–5.0

Cells were treated with adenovirus expression vector encoding human BVR regulated by a Tet promoter (Kravets et al., 2004) and BVR expression was induced by treatment with doxycycline for 16 h. Total RNA was analyzed using a microarray to detect expression of genes involved in cell proliferation. Genes that showed a significant deviation in expression in cells with elevated BVR compared to untreated cells are indicated. Modified from (Kravets et al., 2004).

## BVR Activity in the MAPK Pathway

Environmental stimuli, including growth factors, cytokines and hormones, as well as stress due to heat or altered redox status of the cell (Cobb and Goldsmith, 1995; Jackson and Foster, 2004; Boutros et al., 2008; Kim and Choi, 2010) stimulate receptor complexes (receptor tyrosine kinases, G-protein coupled receptors), that in turn recruit either PI3K or son of sevenless (SOS) and the growth factor bound-2 protein (GRB2), potentially activating two major signaling branches. Constitutively active receptor tyrosine kinases, including those of epidermal growth factor, fibroblast growth factor and platelet-derived growth factor receptors that are frequently found to be mutated or up-regulated in a variety of cancer types. Activation of SOS and GRB2 leads to exchanging Ras-bound GDP for GTP to yield the active form. The activated GTP-Ras complex then interacts with a variety of effector proteins, including members of the Raf kinase family (Vojtek et al., 1993; Geyer and Wittinghofer, 1997). Raf activation in the cell occurs in the presence of activated GTPases, such as the Ras family of proteins, which allow translocation of the Raf protein to the membrane, binding of Raf to Ras (Terai and Matsuda, 2005), and subsequent homodimerization or heterodimerization with the kinase suppressor of Ras (KSR) protein (Brennan et al., 2011). The dimers at this stage are partially active, but transphosphorylate other dimers to give a fully active MAPKKK enzyme (McKay et al., 2011). The Raf proteins then phosphorylate the MAPKK proteins, including MEK1 and MEK2. The activated MEK proteins then phosphorylate the sequence TXY in MAPKs (TEY in ERK1/2) thereby activating these serine/threonine kinases. There are three subfamilies that comprise the MAPK family of protein—the extracellular response kinases (ERK1, ERK2 and the more distantly related ERK5), the p38 MAPKs and the c-Jun N-terminal kinases (JNKs). Phosphorylation of SP or ST sequences in the extensive range of MAPK substrates regulates cellular functions that include metabolism, gene expression, cell proliferation, movement and differentiation, and programmed cell death reviewed in Mebratu and Tesfaigzi (2009).

ERK1 (MW 44 kDa) and ERK2 (42 kDa) share ∼85% sequence identity, with functional of the protein being more highly conserved, and are expressed ubiquitously (Boulton et al., 1991). As would be expected given this high degree of sequence similarity, they largely share the same substrates. Gene knockout studies have indicated that the ERK isoforms have different functions in embryonic development. ERK2 and MEK 1 are essential, and the knockouts show impaired placental development (Giroux et al., 1999; Hatano et al., 2003); by contrast, ERK1 or MEK2 knockouts were phenotypically normal with respect to size, fertility and viability (Pages et al., 1999; Belanger et al., 2003).

In resting cells, ERK is sequestered in the cytoplasm. This sequestration is mediated by multiple mechanisms, including association with MEK (Fukuda et al., 1997), with microtubule structures (Reszka et al., 1995) or with phosphatases, such as the MAP kinase phosphatase MKP3 which complexes with ERK1/2, and by virtue of its NES confines ERK to the cytoplasm (Karlsson et al., 2004). Mitogen stimulation results in rapid (5–10 min) burst of kinase activity, followed by transit to the nucleus, and a more sustained, albeit lower, level of activation that persists through G1 until entry into S-phase (Kahan et al., 1992; Meloche et al., 1992; Meloche, 1995), whereupon ERK is rapidly inactivated. While ERK activity is essential for the G1-S phase transition, it is not sufficient (Kahan et al., 1992). There is evidence to suggest that prolonged activation of ERK might be proapoptotic, whereas transient activation is cytoprotective (di Mari et al., 1999; Arany et al., 2004).

Experiments carried out *in vitro* and in intact cells, indicated that BVR activated both MEK1 and ERK1/2, with the three proteins forming a ternary complex (Lerner-Marmarosh et al., 2008). Over-expressed BVR was almost as effective as IGF-1 in activating ERK1/2 in cultured cells; BVR is itself a substrate for the ERK1/2 kinase activity. Formation of the MEK/ERK/BVR ternary complex was impaired in cells treated with siRNA against BVR, leading to decreased activation of ERK1/2 in response to IGF-1. Although ERK1/2 is not a substrate for BVR kinase activity, the ATP-binding domain of BVR was shown to be necessary for ERK1/2-dependent Elk activation, although ERK1/2 is not phosphorylated by BVR (Lerner-Marmarosh et al., 2008).

Translocation of ERK to the nucleus is essential for its effects on regulating gene transcription, and would therefore be a potential target for mediating its growth promoting properties. ERK1/2 are transcriptional regulators of over 50 genes, including *c-jun*, *c-fos*, *Myc*, NF-κB, *iNOS*, *NR2F*, *Bach1 GATA1*, *STATs*, *HIF1*, and *VEGF* (Giuliani et al., 2004; Ranganathan et al., 2006; Yoon and Seger, 2006; Yazicioglu et al., 2007) that control cell polarity, proliferation, differentiation, adhesion, and invasiveness. We have described BVR-mediated ERK transport to the nucleus, and its inhibition by peptide(s) that disrupt BVR-ERK complex formation and activation of ERK (Lerner-Marmarosh et al., 2008). ERK signaling activates transcription factors including Elk1 that in turn regulate the cell cycle. However, the kinases also inactivate components of the cell death pathways, and stimulate transcription of genes that promote cell survival. Thus phosphorylation by ERK of the FOXO transcription factors promotes their degradation by MDM2-dependent ubiquitination and proteasomal mechanisms. FOXO-dependent transcription targets include antiapoptosis genes such as those encoding Bim or FasL (Burgering and Kops, 2002; Finnberg and El-Deiry, 2004), as well as cell cycle regulators such as cyclin D (Schmidt et al., 2002) and p27/Kip1 (Dijkers et al., 2000). It is noted (Table 1) that p27/Kip1 expression is significantly repressed when BVR is over-expressed. Thus inhibition of ERK activity, e.g., by the C-box peptide, would be expected to inhibit cell proliferation as a consequence of stable FOXO-dependent transcription.

There are two MAPK consensus docking motifs in human BVR: the C-Box (Jacobs et al., 1999) ^162^FGFP and D-Box (Minden and Karin, 1997) ^275^KKRILHCLGL. Both are required for assembly of the ternary complex with MEK1 and ERK2, since BVR bearing mutations in either motif inhibits activation of ERK1/2 in response to IGF-1 treatment, leading to a significant reduction in Elk1-dependent transcriptional activity (Lerner-Marmarosh et al., 2008). Similar observations were made with cells treated with BVR-based peptides bearing the C- or D-Box sequences (Lerner-Marmarosh et al., 2008). ERK1/2 transport into the nucleus was impaired in cells expressing the BVR NLS mutant; likewise, nuclear accumulation of ERK1/2 was observed in cells expressing the NES mutant (Lerner-Marmarosh et al., 2008). Taken together, these observations indicate that BVR is a bidirectional transporter of ERK1/2 between the cytoplasm and nucleus. This is a most significant aspect of BVR’s cellular function, since ERK1/2 relies on transporter proteins to shuttle between the nucleus and cytoplasm, as it does not possess either NLS or NES motifs.

The ribosomal S6 kinase (RSK) family of protein kinases is activated by ERK, resulting in translocation of RSK, at least in part, to the nucleus (Zhao et al., 1996. The proapoptotic BAD protein is phosphorylated by RSK resulting in its inactivation (Bonni et al., 1999, Anjum and Blenis, 2008). In addition, the survival-promoting transcription factor ATF2/CREB is phosphorylated and activated by RSK (Bonni et al., 1999). It is noteworthy in this context that ATF2/CREB is also activated by overexpression of BVR (Kravets et al., 2004; Miralem et al., 2005).

A second, prominent transcription factor target of ERK is c-Myc; phosphorylated c-Myc is stabilized as the modification blocks ubiquitination and hence proteasomal degradation (Sears et al., 2000). cMyc is frequently activated or mutated in an extensive variety of cancer cell types, where it induces expression of genes involved in cell proliferation, including cyclins, CDKs and transcription factors, while simultaneously blocking expression of cell cycle inhibitors (Duronio and Xiong, 2013). It is noteworthy that these genes resemble the cell-cycle genes whose expression was altered by over-expressing BVR (Table 1). It is tempting to speculate that overexpressed BVR targets ERK-dependent c-Myc activation to produce the observed transcriptional profile changes.

In addition, activation of the MAPK family proteins leads to phosphorylation and subsequent polyubiquitination and proteasomal degradation of the pro-apoptotic protein Bim (Hubner et al., 2008). Because phosphorylated Bim is bound less well by the antiapoptotic Bcl2 protein compared to its unphosphorylated form, the net result of its phosphorylation in response to ERK pathway activation is increased cell survival (Ley et al., 2003, 2004). Bcl2 gene expression is enhanced by overexpression of BVR (Kravets et al., 2004). These and other studies are indicative of ERK promotion of resistance to apoptosis; BVR itself has been shown to contribute to resistance to chemically-induced cell death, whether in the context of cancer therapy or oxidative stress inducing compounds such as arsenite (Miralem et al., 2005).

The Ras family comprises some of the earliest identified oncogenes; they were initially described as the transforming gene in oncogenic viruses or in tumors (Malumbres and Barbacid, 2003). Constitutively active Ras mutants that do not exchange bound GTP have been identified in about 30% of all tumors, although they are more prevalent in some tumor types than in others Persistent activation of ERK by mutated Ras or Raf proteins contributes to tumor cell proliferation; indeed the transforming potential of Ras or Raf mutants is absolutely dependent on the downstream MEK and ERK proteins, thus providing an impetus for development of inhibitors of MEK or ERK (Kohno and Pouyssegur, 2003) as therapeutic agents. It is still an unresolved issue as to whether such inhibitors will be useful in the clinical setting. Despite a massive effort in developing such drugs (Chappell et al., 2011), their efficacy in clinical trials has been sadly lacking (Haura et al., 2010). It could be argued that prevention of ERK translocation might be an efficient means of enhancing those functions of ERK that promote cell death, thereby killing tumor cells and controlling cancer development. In normal cells, activated ERK proteins in turn phosphorylate Raf, leading to its inactivation, and quenching the signaling pathway. Breast tumor cells, for example are characterized by dysfunction of the Ras-Raf-MEK-ERK pathway; indeed, the activity of this pathway is increased in one-third of all human cancers (Roskoski, 2012). Activation/phosphorylation of ERK1/2 is correlated with both poor therapeutic response to anti-hormonal drugs and with decreased survival rate in breast cancer patients (Gee et al., 2001).

## BVR, ERK1/2, PKCδ—the Complex

PKCδ functions in multiple signaling pathways, in particular, those that lead to ERK1/2 activation (Gorelik et al., 2007). Moreover, there is evidence that PKCδ activates ERK proteins directly (Ueda et al., 1996). PKCδ is a member of the novel class of PKCs and a key regulator of pathways that control cell growth and proliferation (Steinberg, 2004); however, it also regulates cell division arrest and is involved in glucose signaling (Steinberg, 2008; Newton, 2010). Whether the PKC regulates apoptotic or anti-apoptotic signaling pathways depends on its mode of activation in response to specific stimuli. PKCδ is activated by BVR in autophosphorylation and kinase assays. While PKCδ utilizes BVR as a substrate (Gibbs et al., 2012a), PKCδ was not a substrate for BVR kinase activity *in vitro*. Addition of either wt- or kinase-inactive BVR stimulated PKC activity toward a peptide substrate (Gibbs et al., 2012a). A mutant of PKCδ that lacks part of the pseudosubstrate sequence is constitutively active (Zhao et al., 1998), and this protein was also showed increased activity in the presence of BVR (Miralem et al., 2012). The increased activity of PKCδ was dependent on complex formation with BVR. Proteomic analyses indicated that human BVR was phosphorylated by PKCδ at four serine residues, with two of those sites, Ser^21^ and Ser^230^, being kinetically favored (Miralem et al., 2012). Indeed, a 13 aa peptide that includes Ser^230^ and its flanking sequence was found to be an excellent substrate for PKCδ in *in vitro* assays. Phosphorylation at either Ser^33^ or Ser^237^ was not predicted by computational analysis, and the observed result suggests that they are likely novel PKCδ targets (Miralem et al., 2012).

A complex containing both BVR and PKCδ was observed in immunoprecipitates obtained from either PMA- or IGF-1-treated cells (Gibbs et al., 2012a). *In vivo* association between the proteins was demonstrated by fluorescence resonance energy transfer (FRET). FRET was as detected by fluorescence lifetime imaging microscopy (FLIM); a reduction in fluorescence lifetime in this system indicates that FRET has occurred. Cells cotransfected with EGFP-PKCδ and DsRed2-tagged BVR fusion proteins were treated with either IGF-1 or PMA and reduced fluorescence lifetime was observed in the cotransfected cells compared to cells transfected with EGFP-tagged PKCδ alone, for either IGF-1 or PMA-treated cells (Gibbs et al., 2012a,b); the positive FRET response indicates a close intracellular association of the proteins. A requirement for the human BVR KKRILHCLGLA sequence for interaction with PKCδ was revealed by the observations that a protein carrying mutations in this motif and a peptide with the wt sequence both inhibited the protein: protein interaction. The likelihood is that binding of the PKC to BVR triggers a conformational change in the former to facilitate its activation, thereby positioning BVR for an important role in another pathway leading to tumorigenesis.

The observation that BVR is detected in complexes with ERK1/2 and MEK1/2 (Lerner-Marmarosh et al., 2008), and with PKCδ raised the question of whether BVR and its binding partners might form a larger macromolecular signaling entity. Cells over-expressing both BVR and PKCδ and subsequently stimulated with PMA or IGF-1 were found to include both ERK2 and MEK1 in immunoprecipitates obtained with anti-BVR or anti-PKCδ antibodies (Gibbs et al., 2012a). It is possible that there could be independent cytoplasmic ternary complexes, depending on whether it is MEK1/2 or PKCδ that is activating ERK1/2. Cells were transfected to over express BVR and PKCδ, treated with IGF-1 and complexes were immunoprecipitated from cell lysates with anti-PKCδ antibodies. MEK1 was detected in the immunoprecipitate, together with BVR and ERK2. Since all four proteins were detected in a single immunoprecipitation, it is apparent that an elaborate signaling complex is formed (Gibbs et al., 2012a). Unconstrained activation of ERK by this multiprotein complex is expected to be harmful to the cell, since it would deregulate gene expression and also cell proliferation. Apparently, the complex also contained the protein phosphatase PP2A (Gibbs et al., 2012a) that targets, and inactivates, PKCδ (Srivastava et al., 2002), suggesting a mechanism that would prevent “runaway” signaling by the ERK1/2 complex that essentially involves its self-regulation. As BVR’s kinase activity was not needed for complex formation with PKCδ, BVR is most likely acting as a scaffold to bring the substrate ERK2 into close proximity with its activating PKC or MEK, while maintaining the proteins in their active conformation. Site-directed mutagenesis indicated that complex formation was dependent upon intact BVR C- and D-Box sequences. ERK2 was recruited to the complex by the C-Box, while PKCδ interacted with the D-Box. Treatment of cells with synthetic peptides that include the human BVR C- and D-Box sequences inhibited complex formation. Activation of the transcription factors Elk1 and NF-κB is dependent on ERK activation, while disruption of complex formation by siRNA-mediated depletion of BVR led to attenuated transcriptional activity and expression of reporter genes (Gibbs et al., 2012a). The NF-κB-dependent activation of the iNOS promoter is also dependent on complex formation—ablation of any one of BVR, PKCδ, ERK1/2, or MEK1/2 with the appropriate siRNA suppressed reporter gene expression driven by the iNOS promoter (Gibbs et al., 2012a).

These findings imply a second mechanism by which BVR acts in protection against oxidative stress (Miralem et al., 2005). The significance of BVR/PKCδ binding can be considered in the context of PKC functions in the cell: It is noted that PKCδ is involved in pathways that regulate cell cycle progression resulting in cell proliferation, differentiation or tumorigenesis (Steinberg, 2008; Newton, 2010). Oxidative stress, however, results in PKCδ being a major factor in activation of mitochondrial-linked apoptosis pathways that lead to cell death (Buder-Hoffmann et al., 2009). In this response, caspase3 cleaves the PKC into regulatory and catalytic domains (Steinberg, 2008), and it is the unregulated catalytic domain that is linked to PKCδ-mediated cell death. It is a reasonable suggestion that the BVR/PKCδ complex blocks access of caspase3 to the PKC, thereby attenuating the onset of apoptosis.

**FIGURE 5:**
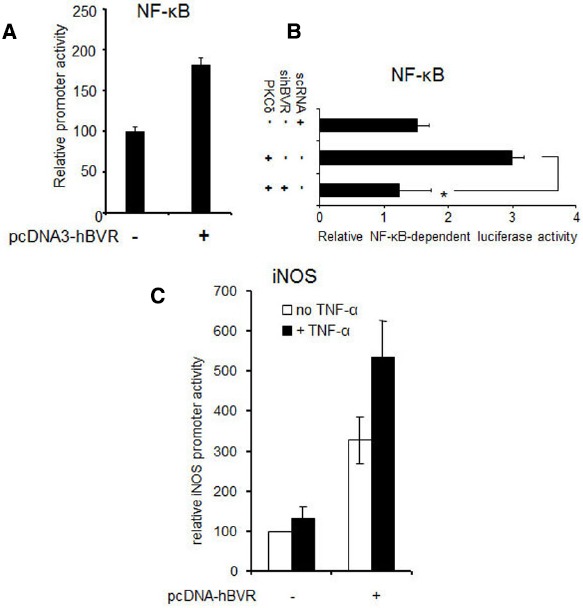
**BVR enhances ERK1/2-dependent transcriptional activation. (A)** NF-κB-dependent promoter activity is stimulated by BVR. Cells were cotransfected with a luciferase reporter plasmid regulated by NF-κB, a β-Gal reporter (to serve as a control for transfection efficiency), and either pcDNA-hBVR or empty vector. 24 h later, NF-κB-dependent promoter activity was measured by luciferase assay and normalized to β-galactosidase. Modified from (Gibbs and Maines, 2007). **(B)** BVR-siRNA suppresses activation of NF-κB by PKCδ. Cells were cotransfected with NF-κB and β-gal reporters, pcDNA-PKCδ and siRNA for BVR or control scRNA. Promoter function was analyzed as in **(A)**, *p < 0.001. Modified from (Gibbs et al., 2012a). **(C)** TNF-α-mediated induction of the iNOS gene is potentiated by BVR. Cells were cotransfected with pcDNA3-hBVR, a luciferase reporter plasmid wherein luciferase is regulated by the *iNOS* promoter, and β-gal reporter. 12 h after DNA addition, cells were treated with TNF-α for a further 12 h. Promoter function was then analyzed as in **(A)**. Modified from (Gibbs and Maines, 2007).

In addition to the hyper-activation of PKCδ and ERK1/2 signals, over-expression of BVR leads to effects on other gene targets. Thus in cells over-expressing BVR, there is a demonstrable increase in the activity of NF-κB, as indicated by expression of reporter genes (Figure 5A). The increased activity is entirely dependent on BVR, as shown by the ability of siRNA against BVR to block PKCδ-dependent activation of NF-κB (Figure 5B). Likewise, tumor necrosis factor-α (TNF-α) dependent induction of transcription of a reporter gene regulated by the promoter for the inducible nitric oxide synthase (iNOS, encoded by the *NOS2* gene) is further increased in cells that over-express BVR (Figure 5C). These data imply that over-expressed BVR is capable of stimulating pro-survival genes.

PKCδ activity is upregulated in breast and lung cancers (Clark et al., 2003; McCracken et al., 2003) and its expression is stimulated by estrogens, thus contributing to resistance to the chemotherapeutic tamoxifen (Cutler et al., 1994; Nabha et al., 2005). Despite the protection afforded by BVR regulating the levels of the antimutagenic bile pigments (Bulmer et al., 2007), BVR is likely to be a contributing factor in the development of cancers, since BVR activates both proteins (Lerner-Marmarosh et al., 2008; Gibbs et al., 2012a; Miralem et al., 2012).

## BVR Modulation of the PI3K/Akt Pathway

The PI3K pathway regulates cell survival (Amaravadi and Thompson, 2005), while in turn, Akt activity is inhibited by ERK (Sinha et al., 2004), so that in primary cells where ERK activation is suppressed, this feedback mechanism enhances cell survival. Conversely, activation of Akt by PMA or EGF inhibits Raf activity and hence the ERK pathway is also inhibited. Nonetheless, overexpression of Akt does not affect ERK phosphorylation (Gervais et al., 2006). ERK phosphorylation in these circumstances might be more dependent on PKCδ (Ueda et al., 1996; Gorelik et al., 2007). However, the activated ERK is sequestered in the cytoplasm by binding to the Akt substrate PEA15, preventing Elk1-dependent transcription and expression of cFos (Gervais et al., 2006). PI3K-Akt signaling controls cell proliferation by regulating the cell cycle as mediated by mTORC1 (Bhaskar and Hay, 2007), phosphorylating GSK3 to inhibit its catalytic activity (Clodfelder-Miller et al., 2005) and by suppressing the cell cycle inhibitors p27 and p21 (Abukhdeir and Park, 2008), thereby stabilizing important cell cycle regulators. Aberrant activation of the pathway is a mechanism for carcinogenesis (Bhaskar and Hay, 2007). As with the MAPK pathway, there is evidence that BVR may regulate key points in this pathway.

It was proposed that BVR might function in the PI3K/Akt pathway based on its two SH2-binding motifs (Maines, 2007). While these are key to interaction with IRK, they should also be able to mediate binding to PI3K. Ischemia/reperfusion injury is characterized by oxidative stress, and in a cell culture model it was noted that phosphorylation of both the regulatory p85 subunit of PI3K and Akt was increased during BVR-dependent HO-1 overexpression, which in turn afforded protection against apoptosis (Pachori et al., 2007). Akt phosphorylation depended on PI3K, which acts upstream of Akt in the signaling pathway, because siRNA-mediated knockdown of PI3K abolished cytoprotection. BVR and the phosphorylated p85 subunit of PI3K were observed to coimmunoprecipitate. Because Akt phosphorylation was also attenuated in cells treated with siRNA directed against BVR, it is likely that BVR is a cytoprotective signaling protein, by virtue of its activation of the PI3K pathway in response to oxidative stress (Pachori et al., 2007). Likewise, upregulation of BVR expression in a human renal proximal tubule cell line was observed after the cells were incubated at low oxygen concentration (Zeng et al., 2008). The BVR promoter is activated during prolonged hypoxia (Gibbs et al., 2010), which is expected to increase BVR protein content in the cell and thus affect its function in cell signaling. In a second model of oxidative stress, it was observed that knockdown of BVR, again by the use of siRNA, rendered cells more vulnerable to sodium arsenite treatment—a higher proportion of apoptotic cells was observed among those treated with siRNA and arsenite compared to those treated with arsenite alone (Miralem et al., 2005). Moreover, renal failure in response to chronic hypoxia results in an epithelial-to-mesenchymal transition (EMT), characterized by decreased expression of E-cadherin and increased expression of vimentin, a diagnostic marker of mesenchymal cells (Zeng et al., 2008). These changes in gene expression were prevented when PI3K activity was inhibited. BVR’s role as a causative factor for these changes is further demonstrated by the observation that its overexpression in normoxic cells led to EMT-like phenotypic changes, and that PI3K inhibition reversed the effects (Zeng et al., 2008). In these cells, PDK1-dependent phosphorylation of Akt was dependent on PI3K activity and BVR was associated with the activated, phosphorylated Akt. Blockade of this association with BVR siRNA effectively prevented EMT, reinforcing the strong connection of BVR to this cascade.

It has been suggested that BVR is expressed on the exterior surface of macrophages (and other cells) where reduction of extracellular biliverdin to bilirubin initiates a cascade mediated by tyrosine phosphorylation of BVR (Wegiel et al., 2009). In turn, phosphorylated BVR binds to the regulatory p85α subunit of PI3K to activate signaling via Akt. Bacterial endotoxin was used to initiate the inflammatory response cultured in macrophages, resulting in a rapid rise in BVR activity. It was suggested that biliverdin mediated its protective effects through induction of synthesis of the anti-inflammatory cytokine, interleukin-10 (IL-10). This up-regulation is partly dependent on activation of Akt; linkage of BVR to Akt-dependent IL-10 synthesis was confirmed by IL-10 expression being blocked by inhibition of Akt activity and by siRNA-mediated attenuation of cell surface BVR. Because BVR is usually considered to be a soluble protein in the cell, the presence of BVR on the plasma membrane poses the question—how did it get there? Transport of BVR to membranes has been associated with activation of BVR in conjunction with PKCs; the conventional and atypical PKCs translocate to the membrane on activation (Newton, 2010, p. 305), and BVR colocalizes with them (Lerner-Marmarosh et al., 2007; Maines et al., 2007). Association with PI3K, as described in Akt activation (Wegiel et al., 2009), is another possibility. In addition, some BVR is associated with caveolae (Kim et al., 2004), which may position it for binding to other membrane proteins.

## Conventional and Atypical PKCs

The PKC proteins provide a means of communication to link signal transduction pathways that respond to extracellular stimuli, and as such they constitute key control points in those pathways (Steinberg, 2008; Newton, 2010). The kinases comprise a family of evolutionarily related proteins that includes three subgroups, known as conventional, novel and atypical PKCs. Each class is differentiated by protein structure, mechanism of activation and function. In addition to the novel PKCδ discussed above, BVR has been shown to activate members of the other PKC classes (Lerner-Marmarosh et al., 2007; Maines et al., 2007; Gibbs et al., 2012a).

### PKCβII

Both isoforms of PKCβ, which arise by an alternate splicing of C-terminal exons, have been implicated in cancer (Garg et al., 2014). As indicated in Figure 1, the insulin and IGF1 signaling pathway is bifurcated. Signals in one arm are transduced via PI3K, while the other proceeds through the MAPKs. The two pathways do regulate each other to some extent—as noted, ERK activation suppresses Akt activity (Sinha et al., 2004), while Akt phosphorylates PEA15, which can then sequester ERK in the cytoplasm (Gervais et al., 2006). Further communication between the two arms is mediated by the conventional PKCs, including PKCβII; oxidative stress is a stimulus for PKC activation (reviewed in Koya and King, 1998; Williams and Tuttle, 2005; Yan et al., 2006). In response to extracellular stimuli that release diacylglycerol and Ca(II), the conventional PKCs bind to receptors for activated C-kinase (RACK) proteins and are translocated to the cell membrane; RACK1 protein includes a series of tandem repeat sequences that are involved in PKC binding (Ron et al., 1994). Both PKCβ and BVR have sequence motifs that resemble the RACK1 repeats, i.e., the pseudo-RACK sequence SVEIWD (Ron and Mochly-Rosen, 1995) and the human BVR motif, ^107^AQELWE. It was proposed, based on these sequence motifs and similar responses of these proteins to oxidative stress that PKCβII and BVR interact in transduction of insulin signals. As described in greater detail elsewhere, BVR activates PKCβII by two discrete mechanisms (Maines et al., 2007; Gibbs et al., 2012b): protein: protein interaction and BVR’s kinase activity.

First, confocal microscopy was used to demonstrate that after stimulation with phorbol12-myistate 13-acetate (PMA), BVR and PKCβII colocalized to the cell membrane (Maines et al., 2007). Additionally, immunoprecipitates from extracts prepared from PMA-treated cells included both proteins, indicating a close physical association that was found to be dependent on both the BVR ATP-binding site and the cysteine-rich, metal binding sequence in BVR’s C-terminus. The nucleotide-binding sequence ^15^GVGRAG was essential for activation of PKCβII, as mutation of BVR Gly^17^ to Ala abolished activation, as did mutating Val^11–14^. The valine residues are not expected to interact directly with PKC-βII as they are not on the exterior of the BVR molecule (Kikuchi et al., 2001; Whitby et al., 2002); it is likely that the Val^11–14^ to Ala^11–14^ mutation destabilizes the BVR secondary structure and thus prevents binding to PKC-βII.

Second, it was demonstrated that BVR was a substrate for PKCβII, and vice versa. This relied on the different divalent metal ion requirements of each kinase. Inclusion of BVR in PKCβII reaction mixtures leads to robust phosphorylation of both proteins (Maines et al., 2007), while addition of BVR to reaction mixtures where the major PKCβII substrate was myelin basic protein (MBP) increased the *V*_max_ of the PKCβII-MBP phosphorylation reaction without altering the *K*_m_. Maturation of PKCβII requires the sequential phosphorylation of three sites, the activation loop, turn and hydrophobic motifs, respectively, located in the C-terminal catalytic domain (Steinberg, 2008; Newton, 2010). BVR was capable of phosphorylating a peptide substrate equivalent to the activation loop of PKCβII, but not peptides based on the turn and hydrophobic motifs. This does not eliminate the possibility of phosphorylation by BVR of sites in the matured PKCβII that could further stimulate its activity.

Because the sequences of the activation loops of PKCs α, β, and γ are very closely related, it is likely that these mechanisms will also apply to the other members of the conventional PKC family (α, βI, and γ), particularly βI, which is derived from an alternate splicing event on the PKCβ mRNA (Newton, 2010).

### PKCζ

Biliverdin reductase also activates the atypical PKC, PKCζ, which has been described as promoting pancreatic tumor growth (Butler et al., 2013), by mechanisms that are similar to the activation of PKCδ. BVR acted as a substrate for PKCζ *in vitro*, with Ser^149^ or Ser^230^ (located in the S/T kinase or an SH2-binding motif, respectively) being the major sites of BVR phosphorylation This PKC was not a substrate for BVR’s kinase activity (Lerner-Marmarosh et al., 2007). Addition of kinase-inactive or wild-type BVR to PKCζ-catalyzed kinase reactions stimulated both autophosphorylation of the PKC and its activity with a specific substrate. It is likely that conformational changes in the PKC caused by its interaction with other proteins leads to its activation in cells. In cells stimulated by TNF-α, BVR and PKCζ formed a complex, as indicated both by their co-immunoprecipitation from cell lysates and by confocal microscopy (Lerner-Marmarosh et al., 2007). Activation of PKCζ activity in the cell depended on the presence of BVR; over-expression of BVR resulted in increased PKCζ activity in response to TNF-α, whereas siRNA treatment significantly inhibited activation. In addition, a peptide containing the PKCζ pseudosubstrate sequence that acts as a competitive inhibitor and siBVR were equally effective in inhibiting the response to TNF-α. Because downstream signaling events that are regulated by this PKC include activation of the AP-1 and NF-κB transcription factors (Moscat et al., 2006), the data indicate that BVR plays a role in cell signaling pathways, distinct from the MAPK and PI3K/Akt pathways, that are nevertheless highly relevant to inflammation and carcinogenesis (Hoesel and Schmid, 2013).

## BVR as an Intracellular Transporter

Biliverdin reductase is detected in almost all cell compartments and transports signaling and regulatory molecules (Lerner-Marmarosh et al., 2007, 2008; Maines et al., 2007; Gibbs et al., 2012a) to both the cell membrane and the nucleus using an energy-dependent process (Tudor et al., 2008). The role of BVR in transporting kinases to the membrane and nucleus has been discussed above. However, it apparent that BVR can also transport heme within the cell, and this process is of importance in regulating transcription of genes that are repressed by the heme- and DNA-binding protein Bach1.

In mammalian cells, binding of heme to Bach1 dissociates the repressor from complexes with small Maf proteins (Igarashi and Sun, 2006) that are associated with specific enhancer regulatory sequences of genes encoding, for example, β-globin and HO-1. This allows replacement of Bach1 transcriptional activators such as NF-E2 p45, or the related Nrf1, Nrf2, and Nrf3, as heterodimers with the small Maf proteins. Fluorescence correlation spectroscopy was used to monitor the diffusion of an EGFP-BVR fusion protein in cells. It was shown that in untreated cells, diffusion constants were approximately equal in the cytoplasm and nucleus (Tudor et al., 2008). In heme-treated cells the rate of BVR diffusion in the nucleus decreased significantly, which suggests that BVR’s interaction with chromatin is enhanced. Heme was demonstrated to bind to the C-terminal seven amino acids of human BVR (Tudor et al., 2008), suggesting that the protein transports heme from the cytoplasm to the nucleus. Treatment of cells with siRNA to ablate BVR expression or expression of mutant BVRs lacking either nuclear import or export sequences blocked HO-1 gene expression after treatment of cells with heme. BVR most likely modulates the signaling network regulated by Maf heterodimers and therefore it is likely to aid in expression of β-globin during differentiation of erythroblasts, and the induction of HO-1 in response to oxidative stress.

## Activation of Transcription

Early response genes, such as c-Fos, c-Jun, ATF2/CREB, and HO-1 are implicated in carcinogenesis (Lopez-Bergami et al., 2010). BVR induces their transcription, whether by activating ERK or more directly. AP1 (Fos-Jun heterodimers) and ATF2/CREB regulate vascular endothelial growth factor (Vleugel et al., 2006), to stimulate angiogenesis, which in turn impacts tumor progression. The primary sequence of human BVR includes a motif that strongly resembles the conserved motif of the b-Zip class of transcription factors, and it also possesses canonical NLS and NES to facilitate transit of the protein between the nuclear and cytoplasmic compartments. In response to known HO-1 inducers, notably bacterial LPS and bromobenzene, and to cGMP, the reductase primarily relocated to the nucleus (Maines et al., 2001), a process requiring a functional NLS. These observations suggested a role in gene regulation, which was subsequently demonstrated in several studies. First, dimeric BVR synthesized in rabbit reticulocyte lysate bound to a DNA fragment that included two activator protein-1 (AP-1) binding sites, derived from the promoter of the mouse HO-1 gene in a gel-shift assay (Ahmad et al., 2002). Mutation of residues in the putative leucine zipper motif or using a target DNA with fewer copies of the AP-1 site abolished binding.

The effect of overexpression of BVR in cells was studied by microarray analyses (Kravets et al., 2004); among the genes upregulated by BVR was that encoding ATF-2/CREB. This transcription factor is a member of the bZip family, and binds to cAMP response elements (CRE). It regulates formation of heterodimers of c-Fos with c-Jun to activate transcription of HO-1 in response to activation of the MAPK pathway (Kietzmann et al., 2003). HO-1 mRNA levels were also increased in these experiments, as was expression of reporter genes regulated by either c-Jun (a component of AP-1) or ATF2. The CRE element was, like the AP-1 elements, a target for BVR binding as revealed by electrophoretic mobility shift. The available data, including the slowed nuclear diffusion of BVR in heme-treated cells (Tudor et al., 2008) indicate that BVR, by binding elements in the HO-1 promoter/enhancer, functions in the cell to regulate HO-1 gene transcription.

It is tempting to speculate that BVR acts as a switch in response to changes in the redox state of the cell; reagents that covalently bind sulfhydryl groups inhibit its activity, whereas reducing agents afford protection (Kutty and Maines, 1981) and its activation is essential for its transcriptional regulation of the stress-responsive HO-1 gene (Kravets et al., 2004; Miralem et al., 2005; Tudor et al., 2008). A series of experiments demonstrated that BVR has a role in the oxidative stress response of the cell. In cells treated with sodium arsenite, a promoter of free radical formation, increased HO-1 expression was dependent on the presence of active BVR in the cell (Miralem et al., 2005). Treatment of cells with siRNA directed against BVR (siBVR) attenuated arsenite-mediated increases in HO-1 expression and c-jun promoter activity. BVR, together with AP-1, was present in DNA:protein complexes in the nuclei of arsenite-treated cells. Moreover, downregulation of BVR by siRNA led to significant increases in the number of apoptotic cells after arsenite treatment, accompanied by increased levels of cellular markers of apoptosis: cytoplasmic cytochrome c, cleavage of poly(ADP-ribose) polymerase and increased expression of the TNF-related apoptosis-inducing ligand (TRAIL) proteins and death receptor-5 mRNA.

## BVR and the Immune System

Development of cancer depends not only on processes occurring within the cancer cell, but on interactions with the extracellular environment. In particular, the immune response of the host organism may protect against the developing tumor, or conversely may provide an environment in which the tumor cells thrive (de Visser et al., 2006). As noted above, elevated expression of BVR was detected in infiltrating T-cells, monocytes, macrophages and lymphocytes seen in human kidney tumors (Maines et al., 1999). Biliverdin has been shown, *inter alia*, to be protective in tissue transplantation, prolonging the survival of xenografts. This process, mediated by NF-κB, is likely due to biliverdin/bilirubin/BVR exerting a dampening effect on the inflammatory response, since markers of inflammation, such as Cox2 and iNOS, as well as inflammatory cytokine expression were significantly decreased in the biliverdin-treated animals (Nakao et al., 2004, 2005). It is noteworthy that BVR is present in caveolae of the plasma membrane (Kim et al., 2004), and also on the external surface of macrophages (Wegiel et al., 2009), suggesting that it may convert extracellular biliverdin to bilirubin. As noted above, BVR activity increased rapidly in response to treatment of macrophages with bacterial lipopolysaccharide, and that extracellular biliverdin was associated with increased production of IL-10, the prototypical anti-inflammatory cytokine (Wegiel et al., 2009). SiRNA against BVR also negated the cytoprotective effects of biliverdin in animal models of shock and acute hepatitis (Wegiel et al., 2009). It was subsequently demonstrated that biliverdin induced phosphorylation of the endothelial nitric oxide synthase (eNOS), which nitrosylates BVR (on cysteine) and induces translocation of BVR to the nucleus (Wegiel et al., 2011). BVR then binds to the AP1 sites of the Toll-like receptor-4 (TLR4) promoter and represses its transcription. This in turn quenches the inflammatory response (Wegiel et al., 2011). The process is completely dependent on eNOS phosphorylation, which is mediated in part by the Ca(II)/calmodulin-dependent kinase. It is noteworthy that chronic inflammation is a potentiating factor in cancer development, and tumor-infiltrating macrophages often predict an unfavorable outcome for cancer patients (de Visser et al., 2006).

There is also some evidence for BVR playing an indirect role in he development of autoimmunity. A yeast two-hybrid assay using BVR as the bait revealed that the Goodpasture antigen-binding protein (GPBP) was a high affinity binding partner (Miralem et al., 2010). Goodpasture syndrome is one of several autoimmune diseases in which GPBP activity is increased, and is characterized by autoimmune recognition of the C-terminal non-collagenous-1 domain of the α3 chain of type IV collagen (Goodpasture antigen) in epithelial basement membranes. The deposition of antibodies in the alveolae results in hemorrhage in the lungs, while their presence in glomerular basement membranes causes a rapidly progressive glomerulonephritis (Saus, 1998; Hudson et al., 2003). GPBP is a non-conventional S/T kinase that recognizes Goodpasture antigen as a substrate (Raya et al., 1999); its expression is regulated by TNF-α (Granero et al., 2005). TNF-α also stimulates the kinase activity of BVR (Lerner-Marmarosh et al., 2007), and it was demonstrated that BVR binds, via its ^281^CX_10_C Zn(II)-binding motif (Maines et al., 1996), to GPBP, to suppress the latter’s kinase activity (Miralem et al., 2010). These observations suggest that BVR suppresses the activity of GPBP, and hence limits the exposure of the epitope of Goodpasture antigen, thus limiting autoimmunity.

## Antioxidant Production

Increased production of ROS has been considered a hallmark of many tumors and cancer cell lines. ROS have long been known to damage proteins, lipids and DNA and are therefore considered to be capable of promoting genomic instability. Cancer cells generate increased levels of ROS, and this in turn stimulates signaling pathways that regulate cell proliferation, metabolic changes and angiogenesis. Biliverdin and bilirubin, the substrate and product of BVR’s reductase activity, have been shown to be antioxidants. It was shown, beginning in the late 1980s, that the micromolar levels of bilirubin at in plasma, arising from normal cellular heme turnover protects cells against oxidative stress-induced injury by acting as a scavenger of ROS (Stocker et al., 1987; Stocker, 2004; Maghzal et al., 2009; Sedlak et al., 2009). At higher concentrations, however, free radicals may be generated by bilirubin; toxicity of bilirubin has been documented since at least the 1950s. Bilirubin is therefore a double-edged sword, being either cytotoxic or cytoprotective, depending on its circulating or tissue concentration, the nature of the stress to the cell, the tissue or cellular target of that stress, and the redox status of the stressed cell reviewed by Kapitulnik (2004). In HeLa cells and mouse embryonic fibroblasts, elevated bilirubin generated oxidative stress in the form of free radicals and ROS (Cesaratto et al., 2007). If serum levels of unconjugated bilirubin are excessive due to increased production and/or decreased conjugation, or the blood-brain barrier is not completely matured, bilirubin enters the brain and binds to specific regions of the brain, notably the basal ganglia. This causes a spectrum of irreversible neurological deficits referred to as “bilirubin encephalopathy” or kernicterus (Shapiro, 2003), with the severity of neurological symptoms depends on the circulating bilirubin concentration. Elevated levels of unconjugated bilirubin in cultured neuronal and astroglial cells results in an array of undesirable metabolic effects, including altered carbohydrate metabolism and uncoupling of mitochondrial oxidative phosphorylation (Kapitulnik, 2004). Inhibition of DNA and protein synthesis, abnormal synthesis and release of neurotransmitters, and apoptosis are also seen (Kapitulnik, 2004).

It is generally accepted that glutathione (GSH) is a prominent endogenous cytoprotective antioxidant; bilirubin has been estimated as providing a similar level of protection, with the two antioxidant systems complementing each other, such that hydrophilic GSH functioning in the aqueous cytosol and nucleus, while the lipophilic bilirubin protects membrane lipids (Sedlak et al., 2009). The cytoprotective functions of bilirubin varies with context; e.g., bilirubin scavenging of free radicals impairs bactericidal activity of neutrophils (Overhaus et al., 2006). In addition, in delayed subarachnoid hemorrhage-induced cerebral vasospasm, high levels of radicals are produced in thromboses at the site of hemorrhage. These radicals oxidize bilirubin generating species that act via PKC-mediated contraction of vascular smooth muscle cells, resulting in chronic vasoconstriction and vasospasm reviewed in (Clark and Sharp, 2006). Relaxation of smooth muscle is also impaired, due to activation of members of the Rho guanosine triphosphatase family leading to inhibition of the myosin light chain phosphatase (Pyne-Geithman et al., 2008).

Despite the adverse effects of elevated bilirubin levels, there is evidence favoring a beneficial role of bilirubin acting as a powerful chain-breaking antioxidant in biological systems (Stocker et al., 1987; Stocker, 2004; Maghzal et al., 2009; Sedlak et al., 2009). It has been demonstrated that cells treated with bilirubin are protected from subsequent treatment with a 10^4^-fold molar excess of hydrogen peroxide (Baranano et al., 2002). Bilirubin thus is an essential contributor to cellular and tissue protection attributed to increased HO-1 production; the increased production and nuclear translocation of HO-1 observed in prostate cancers is likely to be a mechanism of cytoprotection, given the abnormal redox status of tumor cells prior to neovascularization (Sacca et al., 2007; Ferrando et al., 2011, 2013; Biswas et al., 2014).

To account for protective function of bilirubin at what could be considered catalytic concentrations, it has been proposed that there is an amplification cycle in which biliverdin is formed by oxidation of bilirubin and is then reduced by BVR to bilirubin (Sedlak and Snyder, 2004, 2009). Evidence in favor of the cycle included the potency of bilirubin as an antioxidant, while elevated levels of ROS and lipid peroxides which ultimately cause cell death are observed if cellular BVR is depleted (Baranano et al., 2002; Sedlak et al., 2009) However, the observed oxidation of bilirubin to biliverdin is apparently insufficient to support the hypothesis (Maghzal et al., 2009). The discrepancies have been attributed to the different methodologies employed (Sedlak et al., 2009), but there are flaws in the hypothesis that bilirubin can be oxidized to biliverdin. Two *in vitro* reactions between bilirubin and alkyl peroxyl radicals have been suggested (McDonagh, 2010): degradation of bilirubin by peroxyl radicals and oxidation of bilirubin to biliverdin. However, the latter reaction requires serum albumin, is independent of peroxyl radical, and is moreover very inefficient (McDonagh, 2010).

Given the rapid, BVR-catalyzed turnover of biliverdin *in vivo*, those studies that claim that biliverdin itself protects cells should be viewed with some skepticism. Administration of biliverdin to whole animals attenuates injuries that are induced during transplantation in a variety of organs including the small intestine, heart and kidney, thereby prolonging survival of transplant recipients (Nakao et al., 2004, 2005), presumably because of its anti-inflammatory activity. The survival of cardiac allografts is prolonged by treatment with biliverdin, resulting in suppression of both the nuclear factor of activated T-cells (NFAT) and NF-κB activities (Yamashita et al., 2004). In turn, this inhibits expression of inflammatory cytokines and slows T-cell proliferation. It is noted that NF-κB regulates cell proliferation and apoptosis (Perkins and Gilmore, 2006). Treatment of cultured cells treated with biliverdin led to attenuated activation of NF-κB in response to TNF-α (Gibbs and Maines, 2007); biliverdin did not bind directly to NF-κB. Conversely, the transcription of NF-κB-dependent reporters in cells where BVR was over-expressed was significantly enhanced, whether cells were treated with TNF-α or not (Gibbs and Maines, 2007). The inflammatory response depends heavily on the activation of NF-κB, and the activity of TNF-α, suggesting that BVR and biliverdin are themselves modulators of inflammation. As noted, the biliverdin induced Akt signaling and IL-10 expression, mediated by cell-surface BVR and the *in vivo* BVR-dependent cytoprotective effects of biliverdin in animal models of shock and acute hepatitis emphasizes this role (Wegiel et al., 2009). Furthermore, protection by biliverdin against acute liver damage in animal model was found to be dependent on the availability of NO, due to biliverdin/BVR-induced eNOS phosphorylation (Wegiel et al., 2011). *S*-nitrosylated BVR is translocated to the nucleus where it binds to AP-1 sites to block transcription of the TLR4 promoter.

The physiological relevance of bile pigments in cancer development also includes their anti-mutagenic properties; being potent peroxyl radical scavengers, they inhibit several classes of mutagens, including polycyclic aromatic hydrocarbons, heterocyclic amines, and other oxidants (Bulmer et al., 2007). In the Ames test, bilirubin and biliverdin were tested in the presence of known mutagens and found to be anti-mutagenic in the presence of all mutagens examined except sodium azide (Bulmer et al., 2007), nor were the bile pigments themselves mutagenic. Subsequently, it was proposed that exogenous biliverdin and/or bilirubin could be given as dietary supplements; they would be expected to be absorbed via epithelial cells of the intestine, leading to elevated antioxidant concentrations in the circulation. This would be expected to result in a subsequent reduction in the development of cardiovascular disease and cancer (Bulmer et al., 2008), as it is known that both conditions occur at lower frequency in patients with Gilbert Syndrome, which is characterized by elevated levels of circulating bilirubin. This would suggest that administration of bilirubin might be expected to protect against other ROS-mediated pathologies. For example, atherosclerosis is inversely correlated with circulating bilirubin concentration in normal individuals. Ollinger et al. (2007) demonstrated that proliferation of smooth muscle cells of blood vessels was reduced in mice treated with bilirubin (and biliverdin), with the result that narrowing of the vessels was inhibited. Treatment of vascular smooth muscle cells with growth factors *in vitro* displayed cell cycle arrest if they were also treated with bile pigments, a process induced by p53 (Ollinger et al., 2007). Similarly, oral treatment with biliverdin for 4 weeks slowed the progression of glucose intolerance and hyperglycemia in diabetic *db/db* mice (Ikeda et al., 2011), with concomitant increases in expression of the pancreatic and duodenal homeobox 1(Pdx1) transcription factor and insulin content of pancreatic islet cells. Additionally, these changes were accompanied by normalization of oxidative stress markers and in production of NADPH oxidase in pancreatic islets, with a significant reduction in apoptotic cell numbers in the pancreas.

## BVR-based Peptides as Potential Cell Growth Regulators

Biliverdin reductase has emerged as a protein that supports cell growth and proliferation due to its role in supporting MAPK (ERK), PI3K and/or PKC signaling, while also promoting cell survival by generation of bilirubin. One approach to attacking the hyperproliferative and tumorigenic phenotype of cancer cells would be to inhibit the kinase or scaffolding functions of the reductase, or both. This could be achieved by exploiting the numerous functional motifs in the primary structure of the protein. Peptides based on those motifs might be expected to inhibit BVR function, and thereby provide a means of attenuating cell signaling and proliferation.

**FIGURE 6:**
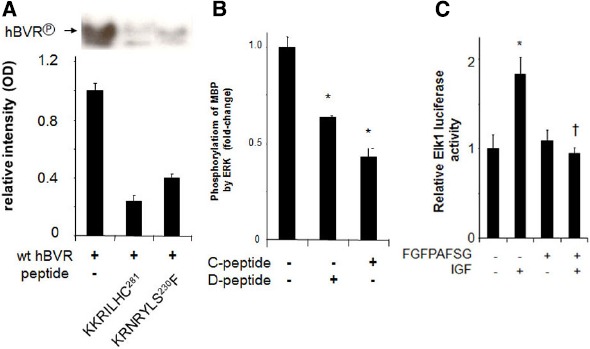
**Peptide inhibition of BVR and ERK1/2. (A)** Autophosphorylation of BVR was determined in reaction mixtures that included no added peptide or either of the BVR-based peptides KKRILHC or KRNRYLSF. The products were detected by autoradiography (top) and quantified by densitometry (bottom). The peptide KRNRYLSF includes a potential IRK target site. **(B)** ERK2 kinase reactions with myelin basic protein as substrate were performed in the presence or absence of the BVR C-Box (FGFPAFSG) and D-Box (KKRILHCLGL) peptides, which inhibit interaction of BVR with ERK, *p < 0.01. **(C)** Peptide FGFPAFSG inhibits ERK-dependent signaling. Cells were transfected with Elk1 expression plasmid and an Elk1-dependent luciferase reporter, then treated with myristoylated peptide for two hours prior to inducing ERK1/2 with IGF-1. After a further 12 h, cells were harvested and luciferase activity was measured. *p < 0.01 compared to untreated control. ^†^*p* < 0.01 compared to IGF-treated.

Regulating the activity of BVR and its ability to form complexes with ERK1/2 or PKCδ could well be a potential approach to treatment of those cancers in which ERK1/2 is activated. In this respect, our studies have offered suggestions that could be exploited. These are illustrated in Figure 6. Human BVR-based peptides have been shown to affect not only BVR activity *per se*, but also the activities of growth promoting kinases that are major players in tumorigenesis and are activated by BVR. The peptides KKRILHC and KRNRYLSF both inhibit the kinase activity of BVR *in vitro* (Figure 6A). Because it has not proven possible to date to separate the kinase and reductase activities of BVR (Salim et al., 2001), both could have some utility in cancer cells, since they would block the formation of bilirubin. It is noted above that the elevated BVR activity in tumors resulted in reduced sensitivity of cells to chemotherapeutic agents (Florczyk et al., 2011). Overcoming this barrier might therefore increase the efficacy of such reagents. Two additional human BVR-based peptides, FGFPAFSG and KKRILHCLGL, have been shown to inhibit the activity of ERK1/2 (Figure 6B). These peptides include the C- (Jacobs et al., 1999) and D-Box (Minden and Karin, 1997) sequences of BVR, and have been shown to inhibit the formation of the complexes including BVR, MEK1, and ERK1/2 (Lerner-Marmarosh et al., 2008) or BVR, PKCδ, and ERK (Miralem et al., 2012). By blocking both upstream activators of ERK, such peptides might be expected to block the activation of downstream targets that lead to cell proliferation. As shown in Figure 6C the FGFPAFSG peptide blocks the activation of ERK-dependent signaling in the cell. Administration of myristoylated peptide is impractical in an intact animal, but other delivery systems, e.g., plasmid expression vectors or nanoparticle systems would be more effective. Another peptide worthy of note is ^230^SFHFKSGSL, a potent PKCδ inhibitor. This peptide was found to enhance PMA-induced apoptosis in cell culture; cells pretreated with peptide for 2 h displayed the membrane blebbing characteristic of apoptosis after a 15 min treatment with PMA (Miralem et al., 2012). The precise mechanisms by which these peptides act are currently unknown. However, two other BVR-based peptides have been demonstrated to bind to IRK and in so doing, alter the secondary structure of the kinase (Gibbs et al., 2014), as demonstrated by fluorescence spectroscopy of dye-tagged IRK. It was noted that the peptides bound to different sites; one of the peptides is both a substrate and inhibitor, with a high affinity for the active site, while the other is an activator that bound outside the active site to alter the secondary structure.

The potential benefits of peptide inhibition of signaling pathways could be offset if they were to display side effects that would compromise BVR activity in otherwise healthy cells, particularly those of macrophages or vascular endothelial cells (Wegiel et al., 2009; Jansen et al., 2010). In both of those systems, biliverdin is a significant signaling component. However, in the macrophages, binding of biliverdin to cell-surface BVR is the initiating event that eventually leads to expression of IL-10. It is not clear whether this involves reduction of biliverdin to bilirubin. Some of the peptides examined to date are inhibitors of BVR reductase or kinase function (Figure 6), but not all. Therefore, off-target effects of peptides might be limited.

The data obtained with the various peptides therefore offer the potential for a novel approach to reversing the detrimental effects of elevated ERK1/2 activity. This would be achieved by inactivating ERK1/2 directly, or by interfering with formation of complexes necessary for its activation, translocation within the cell, or juxtaposition with its targets. It is noteworthy that the existing data suggest that both known activators, MEK in the MAPK signaling pathway and PKCδ (Ueda et al., 1996; Raman et al., 2007) are potentially simultaneous targets of the inhibitors.

## Concluding Remarks

Biliverdin reductase is a cytoprotective and growth promoting protein, as a consequence of both its activation of kinases involved in cell growth, and its being the sole source of the potent anti-oxidant bilirubin. In addition, it directly activates transcription of genes involved in the oxidative stress response. Coupled with increased expression in tumor cells, these properties identify BVR as a tumor promoter. Recent findings have indicated that many of BVR’s growth promoting functions could effectively be negated by use of BVR-based peptides that inhibit its activity and/or its association with growth-promoting kinases. These peptides offer a novel approach to slowing the growth of tumor cells.

### Conflict of Interest Statement

The authors declare that the research was conducted in the absence of any commercial or financial relationships that could be construed as a potential conflict of interest.

## References

[B1] Abukhdeir A. M. Park B. H. (2008). P21 and p27: roles in carcinogenesis and drug resistance. Expert Rev. Mol. Med. 10, e19. 10.1017/S1462399408000744 18590585 PMC2678956

[B2] Ahmad Z. Salim M. Maines M. D. (2002). Human biliverdin reductase is a leucine zipper-like DNA-binding protein and functions in transcriptional activation of heme oxygenase-1 by oxidative stress. J. Biol. Chem. 277, 9226–9232. 10.1074/jbc.M108239200 11773068

[B3] Amaravadi R. Thompson C. B. (2005). The survival kinases Akt and Pim as potential pharmacological targets. J. Clin. Invest. 115, 2618–2624. 10.1172/JCI26273 16200194 PMC1236693

[B4] Anderson E. C. Wong M. H. (2010). Caught in the Akt: regulation of Wnt signaling in the intestine. Gastroenterology 139, 718–722. 10.1053/j.gastro.2010.07.012 20659460 PMC3037729

[B5] Anjum R. Blenis J. (2008). The RSK family of kinases: emerging roles in cellular signalling. Nat. Rev. Mol. Cell Biol. 9, 747–758. 10.1038/nrm2509 18813292

[B6] Arany I. Megyesi J. K. Kaneto H. Price P. M. Safirstein R. L. (2004). Cisplatin-induced cell death is EGFR/src/ERK signaling dependent in mouse proximal tubule cells. Am. J. Physiol. Renal Physiol. 287, F543–F549. 10.1152/ajprenal.00112.2004 15149969

[B7] Baranano D. E. Rao M. Ferris C. D. Snyder S. H. (2002). Biliverdin reductase: a major physiologic cytoprotectant. Proc. Natl. Acad. Sci. U.S.A. 99, 16093–16098. 10.1073/pnas.252626999 12456881 PMC138570

[B8] Belanger L. F. Roy S. Tremblay M. Brott B. Steff A. M. Mourad W. (2003). *Mek2* is dispensable for mouse growth and development. Mol. Cell. Biol. 23, 4778–4787. 10.1128/MCB.23.14.4778-4787.2003 12832465 PMC162209

[B9] Bhaskar P. T. Hay N. (2007). The two TORCs and Akt. Dev. Cell 12, 487–502. 10.1016/j.devcel.2007.03.020 17419990

[B10] Biswas C. Shah N. Muthu M. La P. Fernando A. P. Sengupta S. (2014). Nuclear heme oxygenase-1 (HO-1) modulates subcellular distribution and activation of Nrf2, impacting metabolic and anti-oxidant defenses. J. Biol. Chem. 289, 26882–26894. 10.1074/jbc.M114.567685 25107906 PMC4175329

[B11] Bonni A. Brunet A. West A. E. Datta S. R. Takasu M. A. Greenberg M. E. (1999). Cell survival promoted by the Ras-MAPK signaling pathway by transcription-dependent and -independent mechanisms. Science 286, 1358–1362. 10.1126/science.286.5443.1358 10558990

[B12] Boulton T. G. Nye S. H. Robbins D. J. Ip N. Y. Radziejewska E. Morgenbesser S. D. (1991). ERKs: a family of protein-serine/threonine kinases that are activated and tyrosine phosphorylated in response to insulin and NGF. Cell 65, 663–675. 10.1016/0092-8674(91)90098-J 2032290

[B13] Boutros T. Chevet E. Metrakos P. (2008). Mitogen-activated protein (MAP) kinase/MAP kinase phosphatase regulation: roles in cell growth, death, and cancer. Pharmacol. Rev. 60, 261–310. 10.1124/pr.107.00106 18922965

[B14] Brennan D. F. Dar A. C. Hertz N. T. Chao W. C. Burlingame A. L. Shokat K. M. (2011). A Raf-induced allosteric transition of KSR stimulates phosphorylation of MEK. Nature 472, 366–369. 10.1038/nature09860 21441910

[B15] Buder-Hoffmann S. A. Shukla A. Barrett T. F. Macpherson M. B. Lounsbury K. M. Mossman B. T. (2009). A protein kinase Cδ-dependent protein kinase D pathway modulates ERK1/2 and JNK1/2 phosphorylation and Bim-associated apoptosis by asbestos. Am. J. Pathol. 174, 449–459. 10.2353/ajpath.2009.080180 19116364 PMC2630554

[B16] Bulmer A. C. Blanchfield J. T. Coombes J. S. Toth I. (2008). *In vitro* permeability and metabolic stability of bile pigments and the effects of hydrophilic and lipophilic modification of biliverdin. Bioorg. Med. Chem. 16, 3616–3625. 10.1016/j.bmc.2008.02.008 18304823

[B17] Bulmer A. C. Ried K. Coombes J. S. Blanchfield J. T. Toth I. Wagner K. H. (2007). The anti-mutagenic and antioxidant effects of bile pigments in the Ames *Salmonella* test. Mutat. Res. 629, 122–132. 10.1016/j.mrgentox.2007.01.008 17350329

[B18] Burgering B. M. Kops G. J. (2002). Cell cycle and death control: long live Forkheads. Trends Biochem. Sci. 27, 352–360. 10.1016/S0968-0004(02)02113-8 12114024

[B19] Butler A. M. Scotti Buzhardt M. L. Li S. Smith K. E. Fields A. P. Murray N. R. (2013). Protein kinase C zeta regulates human pancreatic cancer cell transformed growth and invasion through a STAT3-dependent mechanism. PLoS ONE 8:e72061. 10.1371/journal.pone.0072061 24015205 PMC3756013

[B20] Cesaratto L. Calligaris S. D. Vascotto C. Deganuto M. Bellarosa C. Quadrifoglio F. (2007). Bilirubin-induced cell toxicity involves PTEN activation through an APE1/Ref-1-dependent pathway. J. Mol. Med. (Berl.) 85, 1099–1112. 10.1007/s00109-007-0204-3 17479230

[B21] Chappell W. H. Steelman L. S. Long J. M. Kempf R. C. Abrams S. L. Franklin R. A. (2011). Ras/Raf/MEK/ERK and PI3K/PTEN/Akt/mTOR inhibitors: rationale and importance to inhibiting these pathways in human health. Oncotarget 2, 135–164. 21411864 10.18632/oncotarget.240 PMC3260807

[B22] Clark A. S. West K. A. Blumberg P. M. Dennis P. A. (2003). Altered protein kinase C (PKC) isoforms in non-small cell lung cancer cells: PKCδ promotes cellular survival and chemotherapeutic resistance. Cancer Res. 63, 780–786. 12591726

[B23] Clark J. F. Sharp F. R. (2006). Bilirubin oxidation products (BOXes) and their role in cerebral vasospasm after subarachnoid hemorrhage. J. Cereb. Blood Flow Metab. 26, 1223–1233. 10.1038/sj.jcbfm.9600280 16467784

[B24] Clodfelder-Miller B. De Sarno P. Zmijewska A. A. Song L. Jope R. S. (2005). Physiological and pathological changes in glucose regulate brain Akt and glycogen synthase kinase-3. J. Biol. Chem. 280, 39723–39731. 10.1074/jbc.M508824200 16179343 PMC1361688

[B25] Cobb M. H. Goldsmith E. J. (1995). How MAP kinases are regulated. J. Biol. Chem. 270, 14843–14846. 10.1074/jbc.270.25.14843 7797459

[B26] Cutler R. E. Jr. Maizels E. T. Hunzicker-Dunn M. (1994). Delta protein kinase-C in the rat ovary: estrogen regulation and localization. Endocrinology 135, 1669–1678. 7925131 10.1210/endo.135.4.7925131

[B27] de Visser K. E. Eichten A. Coussens L. M. (2006). Paradoxical roles of the immune system during cancer development. Nat. Rev. Cancer 6, 24–37. 10.1038/nrc1782 16397525

[B28] Dijkers P. F. Medema R. H. Pals C. Banerji L. Thomas N. S. Lam E. W. (2000). Forkhead transcription factor FKHR-L1 modulates cytokine-dependent transcriptional regulation of p27(KIP1). Mol. Cell. Biol. 20, 9138–9148. 10.1128/MCB.20.24.9138-9148.2000 11094066 PMC102172

[B29] di Mari J. F. Davis R. Safirstein R. L. (1999). MAPK activation determines renal epithelial cell survival during oxidative injury. Am. J. Physiol. 277, F195–F203. 10444573 10.1152/ajprenal.1999.277.2.F195

[B30] Ding Z. Xu Y. (1994). Purification and properties of cow splenic biliverdin reductase. Prep. Biochem. 24, 193–201. 10.1080/10826069408010093 7831202

[B31] Duronio R. J. Xiong Y. (2013). Signaling pathways that control cell proliferation. Cold Spring Harb. Perspect. Biol. 5, a008904. 10.1101/cshperspect.a008904 23457258 PMC3578363

[B32] Evan G. I. Vousden K. H. (2001). Proliferation, cell cycle and apoptosis in cancer. Nature 411, 342–348. 10.1038/35077213 11357141

[B33] Ferrando M. Gueron G. Elguero B. Giudice J. Salles A. Leskow F. C. (2011). Heme oxygenase 1 (HO-1) challenges the angiogenic switch in prostate cancer. Angiogenesis 14, 467–479. 10.1007/s10456-011-9230-4 21833623

[B34] Ferrando M. Wan X. Meiss R. Yang J. De Siervi A. Navone N. (2013). Heme oxygenase-1 (HO-1) expression in prostate cancer cells modulates the oxidative response in bone cells. PLoS ONE 8:e80315. 10.1371/journal.pone.0080315 24224047 PMC3817116

[B35] Finnberg N. El-Deiry W. S. (2004). Activating FOXO3a, NF-kappaB and p53 by targeting IKKs: an effective multi-faceted targeting of the tumor-cell phenotype? Cancer Biol. Ther. 3, 614–616. 10.4161/cbt.3.7.1057 15254408

[B36] Florczyk U. Golda S. Zieba A. Cisowski J. Jozkowicz A. Dulak J. (2011). Overexpression of biliverdin reductase enhances resistance to chemotherapeutics. Cancer Lett. 300, 40–47. 10.1016/j.canlet.2010.09.003 20934804

[B37] Fukuda M. Gotoh Y. Nishida E. (1997). Interaction of MAP kinase with MAP kinase kinase: its possible role in the control of nucleocytoplasmic transport of MAP kinase. EMBO J. 16, 1901–1908. 10.1093/emboj/16.8.1901 9155016 PMC1169793

[B38] Garg R. Benedetti L. G. Abera M. B. Wang H. Abba M. Kazanietz M. G. (2014). Protein kinase C and cancer: what we know and what we do not. Oncogene 33, 5225–5237. 10.1038/onc.2013.524 24336328 PMC4435965

[B39] Gee J. M. Robertson J. F. Ellis I. O. Nicholson R. I. (2001). Phosphorylation of ERK1/2 mitogen-activated protein kinase is associated with poor response to anti-hormonal therapy and decreased patient survival in clinical breast cancer. Int. J. Cancer 95, 247–254. 10.1002/1097-0215(20010720)95:4<247::AID-IJC1042>3.0.CO;2-S 11400118

[B40] George J. W. Nulk K. Weiss A. Bruss M. L. Cornelius C. E. (1989). Biliverdin reductase activity in cattle, sheep, rabbits and rats. Int. J. Biochem. 21, 477–481. 10.1016/0020-711X(89)90127-4 2759328

[B41] Gervais M. Dugourd C. Muller L. Ardidie C. Canton B. Loviconi L. (2006). Akt down-regulates ERK1/2 nuclear localization and angiotensin II-induced cell proliferation through PEA-15. Mol. Biol. Cell 17, 3940–3951. 10.1091/mbc.E06-06-0501 16822839 PMC1593169

[B42] Geyer M. Wittinghofer A. (1997). GEFs, GAPs, GDIs and effectors: taking a closer (3D) look at the regulation of Ras-related GTP-binding proteins. Curr. Opin. Struct. Biol. 7, 786–792. 10.1016/S0959-440X(97)80147-9 9434896

[B43] Gibbs P. E. Lerner-Marmarosh N. Poulin A. Farah E. Maines M. D. (2014). Human biliverdin reductase-based peptides activate and inhibit glucose uptake through direct interaction with the kinase domain of insulin receptor. FASEB J. 28, 2478–2491. 10.1096/fj.13-247015 24568842 PMC4021440

[B44] Gibbs P. E. Maines M. D. (2007). Biliverdin inhibits activation of NF-kappaB: reversal of inhibition by human biliverdin reductase. Int. J. Cancer 121, 2567–2574. 10.1002/ijc.22978 17683071

[B45] Gibbs P. E. Miralem T. Lerner-Marmarosh N. Tudor C. Maines M. D. (2012a). Formation of ternary complex of human biliverdin reductase-protein kinase Cδ-ERK2 protein is essential for ERK2-mediated activation of Elk1 protein, nuclear factor-κB, and inducible nitric-oxidase synthase (iNOS). J. Biol. Chem. 287, 1066–1079. 10.1074/jbc.M111.279612 22065579 PMC3256887

[B46] Gibbs P. E. Tudor C. Maines M. D. (2012b). Biliverdin reductase: more than a namesake—the reductase, its Peptide fragments, and biliverdin regulate activity of the three classes of protein kinase C. Front. Pharmacol. 3:31. 10.3389/fphar.2012.00031 22419908 PMC3299957

[B47] Gibbs P. E. Miralem T. Maines M. D. (2010). Characterization of the human biliverdin reductase gene structure and regulatory elements: promoter activity is enhanced by hypoxia and suppressed by TNF-α-activated NF-κB. FASEB J. 24, 3239–3254. 10.1096/fj.09-144592 20410444 PMC3231108

[B48] Giroux S. Tremblay M. Bernard D. Cardin-Girard J. F. Aubry S. Larouche L. (1999). Embryonic death of Mek1-deficient mice reveals a role for this kinase in angiogenesis in the labyrinthine region of the placenta. Curr. Biol. 9, 369–372. 10.1016/S0960-9822(99)80164-X 10209122

[B49] Giuliani N. Lunghi P. Morandi F. Colla S. Bonomini S. Hojden M. (2004). Downmodulation of ERK protein kinase activity inhibits VEGF secretion by human myeloma cells and myeloma-induced angiogenesis. Leukemia 18, 628–635. 10.1038/sj.leu.2403269 14737074

[B50] Gorelik G. Fang J. Y. Wu A. Sawalha A. H. Richardson B. (2007). Impaired T cell protein kinase Cδ activation decreases ERK pathway signaling in idiopathic and hydralazine-induced lupus. J. Immunol. 179, 5553–5563. 10.4049/jimmunol.179.8.5553 17911642

[B51] Granero F. Revert F. Revert-Ros F. Lainez S. Martinez-Martinez P. Saus J. (2005). A human-specific TNF-responsive promoter for Goodpasture antigen-binding protein. FEBS J. 272, 5291–5305. 10.1111/j.1742-4658.2005.04925.x 16218959

[B52] Hatano N. Mori Y. Oh-Hora M. Kosugi A. Fujikawa T. Nakai N. (2003). Essential role for ERK2 mitogen-activated protein kinase in placental development. Genes Cells 8, 847–856. 10.1046/j.1365-2443.2003.00680.x 14622137

[B53] Haura E. B. Ricart A. D. Larson T. G. Stella P. J. Bazhenova L. Miller V. A. (2010). A phase II study of PD-0325901, an oral MEK inhibitor, in previously treated patients with advanced non-small cell lung cancer. Clin. Cancer Res. 16, 2450–2457. 10.1158/1078-0432.CCR-09-1920 20332327

[B54] Hellman K. Alaiya A. A. Becker S. Lomnytska M. Schedvins K. Steinberg W. (2009). Differential tissue-specific protein markers of vaginal carcinoma. Br. J. Cancer 100, 1303–1314. 10.1038/sj.bjc.6604975 19367286 PMC2676541

[B55] Hoesel B. Schmid J. A. (2013). The complexity of NF-κB signaling in inflammation and cancer. Mol. Cancer 12, 86. 10.1186/1476-4598-12-86 23915189 PMC3750319

[B56] Hough C. Radu M. Dore J. J. (2012). Tgf-beta induced Erk phosphorylation of smad linker region regulates smad signaling. PLoS ONE 7:e42513. 10.1371/journal.pone.0042513 22880011 PMC3412844

[B57] Huang T. J. Maines M. D. (1990). Bromobenzene-mediated alteration in activity and electrophoretic pattern of biliverdin reductase variants in rat kidney. Mol. Pharmacol. 37, 25–29. 2300045

[B58] Huang T. J. Trakshel G. M. Maines M. D. (1989a). Detection of 10 variants of biliverdin reductase in rat liver by two-dimensional gel electrophoresis. J. Biol. Chem. 264, 7844–7849. 2722768

[B59] Huang T. J. Trakshel G. M. Maines M. D. (1989b). Microheterogeneity of biliverdin reductase in rat liver and spleen: selective suppression of enzyme variants in liver by bromobenzene. Arch. Biochem. Biophys. 274, 617–625. 10.1016/0003-9861(89)90477-3 2802632

[B60] Huang T. J. Trakshel G. M. Maines M. D. (1989c). Multiple forms of biliverdin reductase: modification of the pattern of expression in rat liver by bromobenzene. Arch. Biochem. Biophys. 270, 513–520. 10.1016/0003-9861(89)90533-X 2705777

[B61] Hubner A. Barrett T. Flavell R. A. Davis R. J. (2008). Multisite phosphorylation regulates Bim stability and apoptotic activity. Mol. Cell 30, 415–425. 10.1016/j.molcel.2008.03.025 18498746 PMC2453504

[B62] Hudson B. G. Tryggvason K. Sundaramoorthy M. Neilson E. G. (2003). Alport’s syndrome, Goodpasture’s syndrome, and type IV collagen. N. Engl. J. Med. 348, 2543–2556. 10.1056/NEJMra022296 12815141

[B63] Igarashi K. Sun J. (2006). The heme-Bach1 pathway in the regulation of oxidative stress response and erythroid differentiation. Antioxid. Redox Signal. 8, 107–118. 10.1089/ars.2006.8.107 16487043

[B64] Ikeda N. Inoguchi T. Sonoda N. Fujii M. Takei R. Hirata E. (2011). Biliverdin protects against the deterioration of glucose tolerance in db/db mice. Diabetologia 54, 2183–2191. 10.1007/s00125-011-2197-2 21614569

[B65] Inman G. J. (2011). Switching TGFβ from a tumor suppressor to a tumor promoter. Curr. Opin. Genet. Dev. 21, 93–99. 10.1016/j.gde.2010.12.004 21251810

[B66] Jackson D. N. Foster D. A. (2004). The enigmatic protein kinase Cδ: complex roles in cell proliferation and survival. FASEB J. 18, 627–636. 10.1096/fj.03-0979rev 15054085

[B67] Jacobs D. Glossip D. Xing H. Muslin A. J. Kornfeld K. (1999). Multiple docking sites on substrate proteins form a modular system that mediates recognition by ERK MAP kinase. Genes Dev. 13, 163–175. 10.1101/gad.13.2.163 9925641 PMC316390

[B68] Jansen T. Hortmann M. Oelze M. Opitz B. Steven S. Schell R. (2010). Conversion of biliverdin to bilirubin by biliverdin reductase contributes to endothelial cell protection by heme oxygenase-1-evidence for direct and indirect antioxidant actions of bilirubin. J. Mol. Cell. Cardiol. 49, 186–195. 10.1016/j.yjmcc.2010.04.011 20430037

[B69] Jiang J. Hui C. C. (2008). Hedgehog signaling in development and cancer. Dev. Cell 15, 801–812. 10.1016/j.devcel.2008.11.010 19081070 PMC6443374

[B70] Kahan C. Seuwen K. Meloche S. Pouyssegur J. (1992). Coordinate, biphasic activation of p44 mitogen-activated protein kinase and S6 kinase by growth factors in hamster fibroblasts. Evidence for thrombin-induced signals different from phosphoinositide turnover and adenylylcyclase inhibition. J. Biol. Chem. 267, 13369–13375. 1320018

[B71] Kapitulnik J. (2004). Bilirubin: an endogenous product of heme degradation with both cytotoxic and cytoprotective properties. Mol. Pharmacol. 66, 773–779. 10.1124/mol.104.002832 15269289

[B72] Kapitulnik J. Maines M. D. (2009). Pleiotropic functions of biliverdin reductase: cellular signaling and generation of cytoprotective and cytotoxic bilirubin. Trends Pharmacol. Sci. 30, 129–137. 10.1016/j.tips.2008.12.003 19217170

[B73] Karlsson M. Mathers J. Dickinson R. J. Mandl M. Keyse S. M. (2004). Both nuclear-cytoplasmic shuttling of the dual specificity phosphatase MKP-3 and its ability to anchor MAP kinase in the cytoplasm are mediated by a conserved nuclear export signal. J. Biol. Chem. 279, 41882–41891. 10.1074/jbc.M406720200 15269220

[B74] Kietzmann T. Samoylenko A. Immenschuh S. (2003). Transcriptional regulation of heme oxygenase-1 gene expression by MAP kinases of the JNK and p38 pathways in primary cultures of rat hepatocytes. J. Biol. Chem. 278, 17927–17936. 10.1074/jbc.M203929200 12637567

[B75] Kikuchi A. Park S. Y. Miyatake H. Sun D. Sato M. Yoshida T. (2001). Crystal structure of rat biliverdin reductase. Nat. Struct. Biol. 8, 221–225. 10.1038/84955 11224565

[B76] Kim E. K. Choi E. J. (2010). Pathological roles of MAPK signaling pathways in human diseases. Biochim. Biophys. Acta 1802, 396–405. 10.1016/j.bbadis.2009.12.009 20079433

[B77] Kim H. P. Wang X. Galbiati F. Ryter S. W. Choi A. M. (2004). Caveolae compartmentalization of heme oxygenase-1 in endothelial cells. FASEB J. 18, 1080–1089. 10.1096/fj.03-1391com 15226268

[B78] Kim S. S. Seong S. Lim S. H. Kim S. Y. (2013). Biliverdin reductase plays a crucial role in hypoxia-induced chemoresistance in human glioblastoma. Biochem. Biophys. Res. Commun. 440, 658–663. 10.1016/j.bbrc.2013.09.120 24113378

[B79] Klaus A. Birchmeier W. (2008). Wnt signalling and its impact on development and cancer. Nat. Rev. Cancer 8, 387–398. 10.1038/nrc2389 18432252

[B80] Kohno M. Pouyssegur J. (2003). Pharmacological inhibitors of the ERK signaling pathway: application as anticancer drugs. Prog. Cell Cycle Res. 5, 219–224. 14593716

[B81] Koya D. King G. L. (1998). Protein kinase C activation and the development of diabetic complications. Diabetes Metab. Res. Rev. 47, 859–866. 10.2337/diabetes.47.6.859 9604860

[B82] Kravets A. Hu Z. Miralem T. Torno M. D. Maines M. D. (2004). Biliverdin reductase, a novel regulator for induction of activating transcription factor-2 and heme oxygenase-1. J. Biol. Chem. 279, 19916–19923. 10.1074/jbc.M314251200 14988408

[B83] Kutty R. K. Maines M. D. (1981). Purification and characterization of biliverdin reductase from rat liver. J. Biol. Chem. 256, 3956–3962. 7217067

[B84] Leber M. F. Efferth T. (2009). Molecular principles of cancer invasion and metastasis (review). Int. J. Oncol. 34, 881–895. 10.3892/ijo_00000214 19287945

[B85] Lerner-Marmarosh N. Miralem T. Gibbs P. E. Maines M. D. (2007). Regulation of TNF-α-activated PKC-ζ signaling by the human biliverdin reductase: identification of activating and inhibitory domains of the reductase. FASEB J. 21, 3949–3962. 10.1096/fj.07-8544com 17639074

[B86] Lerner-Marmarosh N. Miralem T. Gibbs P. E. Maines M. D. (2008). Human biliverdin reductase is an ERK activator; hBVR is an ERK nuclear transporter and is required for MAPK signaling. Proc. Natl. Acad. Sci. U.S.A. 105, 6870–6875. 10.1073/pnas.0800750105 18463290 PMC2383961

[B87] Lerner-Marmarosh N. Shen J. Torno M. D. Kravets A. Hu Z. Maines M. D. (2005). Human biliverdin reductase: a member of the insulin receptor substrate family with serine/threonine/tyrosine kinase activity. Proc. Natl. Acad. Sci. U.S.A. 102, 7109–7114. 10.1073/pnas.0502173102 15870194 PMC1088173

[B88] Levett D. Z. Vigano A. Capitanio D. Vasso M. De Palma S. Moriggi M. (2014). Changes in muscle proteomics in the course of the Caudwell Research Expedition to Mt. Everest. Proteomics 15, 160–171. 10.1002/pmic.201400306 25370915

[B89] Ley R. Balmanno K. Hadfield K. Weston C. Cook S. J. (2003). Activation of the ERK1/2 signaling pathway promotes phosphorylation and proteasome-dependent degradation of the BH3-only protein, Bim. J. Biol. Chem. 278, 18811–18816. 10.1074/jbc.M301010200 12646560

[B90] Ley R. Ewings K. E. Hadfield K. Howes E. Balmanno K. Cook S. J. (2004). Extracellular signal-regulated kinases 1/2 are serum-stimulated “Bim(EL) kinases” that bind to the BH3-only protein Bim(EL) causing its phosphorylation and turnover. J. Biol. Chem. 279, 8837–8847. 10.1074/jbc.M311578200 14681225

[B91] Lopez-Bergami P. Lau E. Ronai Z. (2010). Emerging roles of ATF2 and the dynamic AP1 network in cancer. Nat. Rev. Cancer 10, 65–76. 10.1038/nrc2681 20029425 PMC2874064

[B92] Maghzal G. J. Leck M. C. Collinson E. Li C. Stocker R. (2009). Limited role for the bilirubin-biliverdin redox amplification cycle in the cellular antioxidant protection by biliverdin reductase. J. Biol. Chem. 284, 29251–29259. 10.1074/jbc.M109.037119 19690164 PMC2785555

[B93] Maines M. D. (2005). New insights into biliverdin reductase functions: linking heme metabolism to cell signaling. Physiology (Bethesda) 20, 382–389. 10.1152/physiol.00029.2005 16287987

[B94] Maines M. D. (2007). Biliverdin reductase: PKC interaction at the cross-talk of MAPK and PI3K signaling pathways. Antioxid. Redox Signal. 9, 2187–2195. 10.1089/ars.2007.1805 17919068

[B95] Maines M. D. Abrahamsson P. A. (1996). Expression of heme oxygenase-1 (HSP32) in human prostate: normal, hyperplastic, and tumor tissue distribution. Urology 47, 727–733. 10.1016/S0090-4295(96)00010-6 8650873

[B96] Maines M. D. Ewing J. F. Huang T. J. Panahian N. (2001). Nuclear localization of biliverdin reductase in the rat kidney: response to nephrotoxins that induce heme oxygenase-1. J. Pharmacol. Exp. Ther. 296, 1091–1097. 11181945

[B97] Maines M. D. Mayer R. D. Erturk E. Huang T. J. Disantagnese A. (1999). The oxidoreductase, biliverdin reductase, is induced in human renal carcinoma—pH and cofactor-specific increase in activity. J. Urol. 162, 1467–1472. 10.1016/S0022-5347(05)68342-5 10492239

[B98] Maines M. D. Miralem T. Lerner-Marmarosh N. Shen J. Gibbs P. E. (2007). Human biliverdin reductase, a previously unknown activator of protein kinase C βII. J. Biol. Chem. 282, 8110–8122. 10.1074/jbc.M513427200 17227757

[B99] Maines M. D. Polevoda B. V. Huang T. J. Mccoubrey W. K. Jr. (1996). Human biliverdin IXα reductase is a zinc-metalloprotein. Characterization of purified and *Escherichia coli* expressed enzymes. Eur. J. Biochem. 235, 372–381. 10.1111/j.1432-1033.1996.00372.x 8631357

[B100] Malumbres M. Barbacid M. (2003). RAS oncogenes: the first 30 years. Nat. Rev. Cancer 3, 459–465. 10.1038/nrc1097 12778136

[B101] Massague J. (2008). TGFβ in Cancer. Cell 134, 215–230. 10.1016/j.cell.2008.07.001 18662538 PMC3512574

[B102] McCracken M. A. Miraglia L. J. Mckay R. A. Strobl J. S. (2003). Protein kinase Cδ is a prosurvival factor in human breast tumor cell lines. Mol. Cancer Ther. 2, 273–281. 12657722

[B103] McDonagh A. F. (2010). The biliverdin-bilirubin antioxidant cycle of cellular protection: missing a wheel? Free Radic. Biol. Med. 49, 814–820. 10.1016/j.freeradbiomed.2010.06.001 20547221

[B104] McKay M. M. Freeman A. K. Morrison D. K. (2011). Complexity in KSR function revealed by Raf inhibitor and KSR structure studies. Small GTPases 2, 276–281. 10.4161/sgtp.2.5.17740 22292131 PMC3265819

[B105] Mebratu Y. Tesfaigzi Y. (2009). How ERK1/2 activation controls cell proliferation and cell death: is subcellular localization the answer? Cell Cycle 8, 1168–1175. 10.4161/cc.8.8.8147 19282669 PMC2728430

[B106] Meloche S. (1995). Cell cycle reentry of mammalian fibroblasts is accompanied by the sustained activation of p44mapk and p42mapk isoforms in the G1 phase and their inactivation at the G1/S transition. J. Cell. Physiol. 163, 577–588. 10.1002/jcp.1041630319 7775600

[B107] Meloche S. Seuwen K. Pages G. Pouyssegur J. (1992). Biphasic and synergistic activation of p44mapk (ERK1) by growth factors: correlation between late phase activation and mitogenicity. Mol. Endocrinol. 6, 845–854. 1603090 10.1210/mend.6.5.1603090

[B108] Minden A. Karin M. (1997). Regulation and function of the JNK subgroup of MAP kinases. Biochim. Biophys. Acta 1333, F85–F104. 10.1016/s0304-419x(97)00018-8 9395283

[B109] Miralem T. Gibbs P. E. Revert F. Saus J. Maines M. D. (2010). Human biliverdin reductase suppresses Goodpasture antigen-binding protein (GPBP) kinase activity: the reductase regulates tumor necrosis factor-α-NF-κB-dependent GPBP expression. J. Biol. Chem. 285, 12551–12558. 10.1074/jbc.M109.032771 20177069 PMC2857060

[B110] Miralem T. Hu Z. Torno M. D. Lelli K. M. Maines M. D. (2005). Small interference RNA-mediated gene silencing of human biliverdin reductase, but not that of heme oxygenase-1, attenuates arsenite-mediated induction of the oxygenase and increases apoptosis in 293A kidney cells. J. Biol. Chem. 280, 17084–17092. 10.1074/jbc.M413121200 15741166

[B111] Miralem T. Lerner-Marmarosh N. Gibbs P. E. Tudor C. Hagen F. K. Maines M. D. (2012). The human biliverdin reductase-based peptide fragments and biliverdin regulate protein kinase Cδ activity: the peptides are inhibitors or substrate for the protein kinase C. J. Biol. Chem. 287, 24698–24712. 10.1074/jbc.M111.326504 22584576 PMC3397897

[B112] Moscat J. Rennert P. Diaz-Meco M. T. (2006). PKCζ at the crossroad of NF-κB and Jak1/Stat6 signaling pathways. Cell Death. Differ. 13, 702–711. 10.1038/sj.cdd.4401823 16322752

[B113] Myers M. G. Jr. Zhang Y. Aldaz G. A. Grammer T. Glasheen E. M. (1996). YMXM motifs and signaling by an insulin receptor substrate 1 molecule without tyrosine phosphorylation sites. Mol. Cell. Biol. 16, 4147–4155. 8754813 10.1128/mcb.16.8.4147 PMC231411

[B114] Nabha S. M. Glaros S. Hong M. Lykkesfeldt A. E. Schiff R. Osborne K. (2005). Upregulation of PKC-δ contributes to antiestrogen resistance in mammary tumor cells. Oncogene 24, 3166–3176. 10.1038/sj.onc.1208502 15735693

[B115] Nakao A. Neto J. S. Kanno S. Stolz D. B. Kimizuka K. Liu F. (2005). Protection against ischemia/reperfusion injury in cardiac and renal transplantation with carbon monoxide, biliverdin and both. Am. J. Transplant. 5, 282–291. 10.1111/j.1600-6143.2004.00695.x 15643987

[B116] Nakao A. Otterbein L. E. Overhaus M. Sarady J. K. Tsung A. Kimizuka K. (2004). Biliverdin protects the functional integrity of a transplanted syngeneic small bowel. Gastroenterology 127, 595–606. 10.1053/j.gastro.2004.05.059 15300591

[B117] Newton A. C. (1995). Protein kinase C. Seeing two domains. Curr. Biol. 5, 973–976. 10.1016/S0960-9822(95)00191-6 8542286

[B118] Newton A. C. (2010). Protein kinase C: poised to signal. Am. J. Physiol. Endocrinol. Metab. 298, E395–E402. 10.1152/ajpendo.00477.2009 19934406 PMC2838521

[B119] Ollinger R. Yamashita K. Bilban M. Erat A. Kogler P. Thomas M. (2007). Bilirubin and biliverdin treatment of atherosclerotic diseases. Cell Cycle 6, 39–43. 10.4161/cc.6.1.3700 17245120

[B120] Overhaus M. Moore B. A. Barbato J. E. Behrendt F. F. Doering J. G. Bauer A. J. (2006). Biliverdin protects against polymicrobial sepsis by modulating inflammatory mediators. Am. J. Physiol. Gastrointest. Liver Physiol. 290, G695–G703. 10.1152/ajpgi.00152.2005 16537973

[B121] Pachori A. S. Smith A. Mcdonald P. Zhang L. Dzau V. J. Melo L. G. (2007). Heme-oxygenase-1-induced protection against hypoxia/reoxygenation is dependent on biliverdin reductase and its interaction with PI3K/Akt pathway. J. Mol. Cell. Cardiol. 43, 580–592. 10.1016/j.yjmcc.2007.08.003 17920074 PMC2699998

[B122] Pages G. Guerin S. Grall D. Bonino F. Smith A. Anjuere F. (1999). Defective thymocyte maturation in p44 MAP kinase (Erk 1) knockout mice. Science 286, 1374–1377. 10.1126/science.286.5443.1374 10558995

[B123] Pallua J. D. Schaefer G. Seifarth C. Becker M. Meding S. Rauser S. (2013). MALDI-MS tissue imaging identification of biliverdin reductase B overexpression in prostate cancer. J. Proteomics 91, 500–514. 10.1016/j.jprot.2013.08.003 23954705

[B124] Perkins N. D. Gilmore T. D. (2006). Good cop, bad cop: the different faces of NF-κB. Cell Death Differ. 13, 759–772. 10.1038/sj.cdd.4401838 16410803

[B125] Pyne-Geithman G. J. Nair S. G. Caudell D. N. Clark J. F. (2008). PKC and Rho in vascular smooth muscle: activation by BOXes and SAH CSF. Front. Biosci. 13:1526–1534. 10.2741/2778 17981646 PMC2430991

[B126] Raman M. Chen W. Cobb M. H. (2007). Differential regulation and properties of MAPKs. Oncogene 26, 3100–3112. 10.1038/sj.onc.1210392 17496909

[B127] Ranganathan A. Yazicioglu M. N. Cobb M. H. (2006). The nuclear localization of ERK2 occurs by mechanisms both independent of and dependent on energy. J. Biol. Chem. 281, 15645–15652. 10.1074/jbc.M513866200 16595679

[B128] Raya A. Revert F. Navarro S. Saus J. (1999). Characterization of a novel type of serine/threonine kinase that specifically phosphorylates the human goodpasture antigen. J. Biol. Chem. 274, 12642–12649. 10.1074/jbc.274.18.12642 10212244

[B129] Reszka A. A. Seger R. Diltz C. D. Krebs E. G. Fischer E. H. (1995). Association of mitogen-activated protein kinase with the microtubule cytoskeleton. Proc. Natl. Acad. Sci. U.S.A. 92, 8881–8885. 10.1073/pnas.92.19.8881 7568036 PMC41071

[B130] Rigney E. Mantle T. J. (1988). The reaction mechanism of bovine kidney biliverdin reductase. Biochim. Biophys. Acta 957, 237–242. 10.1016/0167-4838(88)90278-6 3191141

[B131] Ron D. Chen C. H. Caldwell J. Jamieson L. Orr E. Mochly-Rosen D. (1994). Cloning of an intracellular receptor for protein kinase C: a homolog of the beta subunit of G proteins. Proc. Natl. Acad. Sci. U.S.A. 91, 839–843. 10.1073/pnas.91.3.839 8302854 PMC521407

[B132] Ron D. Mochly-Rosen D. (1995). An autoregulatory region in protein kinase C: the pseudoanchoring site. Proc. Natl. Acad. Sci. U.S.A. 92, 492–496. 10.1073/pnas.92.2.492 7831317 PMC42767

[B133] Roskoski R. Jr. (2012). ERK1/2 MAP kinases: structure, function, and regulation. Pharmacol. Res. 66, 105–143. 10.1016/j.phrs.2012.04.005 22569528

[B134] Sacca P. Meiss R. Casas G. Mazza O. Calvo J. C. Navone N. (2007). Nuclear translocation of haeme oxygenase-1 is associated to prostate cancer. Br. J. Cancer 97, 1683–1689. 10.1038/sj.bjc.6604081 18026199 PMC2360287

[B135] Salim M. Brown-Kipphut B. A. Maines M. D. (2001). Human biliverdin reductase is autophosphorylated, and phosphorylation is required for bilirubin formation. J. Biol. Chem. 276, 10929–10934. 10.1074/jbc.M010753200 11278740

[B136] Saus J. (1998). Goodpasture’s Syndrome. London: Academic Press, Ltd.

[B137] Schluchter W. M. Glazer A. N. (1997). Characterization of cyanobacterial biliverdin reductase. Conversion of biliverdin to bilirubin is important for normal phycobiliprotein biosynthesis. J. Biol. Chem. 272, 13562–13569. 10.1074/jbc.272.21.13562 9153203

[B138] Schmidt M. Fernandez De Mattos S. Van Der Horst A. Klompmaker R. Kops G. J. Lam E. W. (2002). Cell cycle inhibition by FoxO forkhead transcription factors involves downregulation of cyclin D. Mol. Cell. Biol. 22, 7842–7852. 10.1128/MCB.22.22.7842-7852.2002 12391153 PMC134724

[B139] Sears R. Nuckolls F. Haura E. Taya Y. Tamai K. Nevins J. R. (2000). Multiple Ras-dependent phosphorylation pathways regulate Myc protein stability. Genes Dev. 14, 2501–2514. 10.1101/gad.836800 11018017 PMC316970

[B140] Sedlak T. W. Saleh M. Higginson D. S. Paul B. D. Juluri K. R. Snyder S. H. (2009). Bilirubin and glutathione have complementary antioxidant and cytoprotective roles. Proc. Natl. Acad. Sci. U.S.A. 106, 5171–5176. 10.1073/pnas.0813132106 19286972 PMC2664041

[B141] Sedlak T. W. Snyder S. H. (2004). Bilirubin benefits: cellular protection by a biliverdin reductase antioxidant cycle. Pediatrics 113, 1776–1782. 10.1542/peds.113.6.1776 15173506

[B142] Sedlak T. W. Snyder S. H. (2009). Cycling the wagons for biliverdin reductase. J. Biol. Chem. 284, le11. 10.1074/jbc.L109.037119 19897493 PMC2786007

[B143] Sethi N. Kang Y. (2011). Notch signalling in cancer progression and bone metastasis. Br. J. Cancer 105, 1805–1810. 10.1038/bjc.2011.497 22075946 PMC3251892

[B144] Shapiro S. M. (2003). Bilirubin toxicity in the developing nervous system. Pediatr. Neurol. 29, 410–421. 10.1016/j.pediatrneurol.2003.09.011 14684236

[B145] Singleton J. W. Laster L. (1965). Biliverdin reductase of guinea pig liver. J. Biol. Chem. 240, 4780–4789. 4378982

[B146] Sinha D. Bannergee S. Schwartz J. H. Lieberthal W. Levine J. S. (2004). Inhibition of ligand-independent ERK1/2 activity in kidney proximal tubular cells deprived of soluble survival factors up-regulates Akt and prevents apoptosis. J. Biol. Chem. 279, 10962–10972. 10.1074/jbc.M312048200 14701865

[B147] Song S. Wang S. Ma J. Yao L. Xing H. Zhang L. (2013). Biliverdin reductase/bilirubin mediates the anti-apoptotic effect of hypoxia in pulmonary arterial smooth muscle cells through ERK1/2 pathway. Exp. Cell Res. 319, 1973–1987. 10.1016/j.yexcr.2013.05.015 23722043

[B148] Srivastava J. Goris J. Dilworth S. M. Parker P. J. (2002). Dephosphorylation of PKCδ by protein phosphatase 2Ac and its inhibition by nucleotides. FEBS Lett. 516, 265–269. 10.1016/S0014-5793(02)02500-0 11959144

[B149] Steinberg S. F. (2004). Distinctive activation mechanisms and functions for protein kinase Cδ. Biochem. J. 384, 449–459. 10.1042/BJ20040704 15491280 PMC1134130

[B150] Steinberg S. F. (2008). Structural basis of protein kinase C isoform function. Physiol. Rev. 88, 1341–1378. 10.1152/physrev.00034.2007 18923184 PMC2899688

[B151] Stocker R. (2004). Antioxidant activities of bile pigments. Antioxid. Redox Signal. 6, 841–849. 10.1089/ars.2004.6.841 15345144

[B152] Stocker R. Yamamoto Y. Mcdonagh A. F. Glazer A. N. Ames B. N. (1987). Bilirubin is an antioxidant of possible physiological importance. Science 235, 1043–1046. 10.1126/science.3029864 3029864

[B153] Tenhunen R. Ross M. E. Marver H. S. Schmid R. (1970). Reduced nicotinamide-adenine dinucleotide phosphate dependent biliverdin reductase: partial purification and characterization. Biochemistry 9, 298–303. 10.1021/bi00804a016 4391687

[B154] Terai K. Matsuda M. (2005). Ras binding opens c-Raf to expose the docking site for mitogen-activated protein kinase kinase. EMBO Rep. 6, 251–255. 10.1038/sj.embor.7400349 15711535 PMC1299259

[B155] Tudor C. Lerner-Marmarosh N. Engelborghs Y. Gibbs P. E. Maines M. D. (2008). Biliverdin reductase is a transporter of haem into the nucleus and is essential for regulation of HO-1 gene expression by haematin. Biochem. J. 413, 405–416. 10.1042/BJ20080018 18412543 PMC2723824

[B156] Ueda Y. Hirai S. Osada S. Suzuki A. Mizuno K. Ohno S. (1996). Protein kinase C activates the MEK-ERK pathway in a manner independent of Ras and dependent on Raf. J. Biol. Chem. 271, 23512–23519. 10.1074/jbc.271.38.23512 8798560

[B157] Vleugel M. M. Greijer A. E. Bos R. Van Der Wall E. Van Diest P. J. (2006). c-Jun activation is associated with proliferation and angiogenesis in invasive breast cancer. Hum. Pathol. 37, 668–674. 10.1016/j.humpath.2006.01.022 16733206

[B158] Vojtek A. B. Hollenberg S. M. Cooper J. A. (1993). Mammalian Ras interacts directly with the serine/threonine kinase Raf. Cell 74, 205–214. 10.1016/0092-8674(93)90307-C 8334704

[B159] Wegiel B. Baty C. J. Gallo D. Csizmadia E. Scott J. R. Akhavan A. (2009). Cell surface biliverdin reductase mediates biliverdin-induced anti-inflammatory effects via phosphatidylinositol 3-kinase and Akt. J. Biol. Chem. 284, 21369–21378. 10.1074/jbc.M109.027433 19509285 PMC2755861

[B160] Wegiel B. Gallo D. Csizmadia E. Roger T. Kaczmarek E. Harris C. (2011). Biliverdin inhibits Toll-like receptor-4 (TLR4) expression through nitric oxide-dependent nuclear translocation of biliverdin reductase. Proc. Natl. Acad. Sci. U.S.A. 108, 18849–18854. 10.1073/pnas.1108571108 22042868 PMC3219137

[B161] Whitby F. G. Phillips J. D. Hill C. P. Mccoubrey W. Maines M. D. (2002). Crystal structure of a biliverdin IXalpha reductase enzyme-cofactor complex. J. Mol. Biol. 319, 1199–1210. 10.1016/S0022-2836(02)00383-2 12079357

[B162] Williams M. E. Tuttle K. R. (2005). The next generation of diabetic nephropathy therapies: an update. Adv. Chronic Kidney Dis. 12, 212–222. 10.1053/j.ackd.2005.01.011 15822057

[B163] Yamashita K. Mcdaid J. Ollinger R. Tsui T. Y. Berberat P. O. Usheva A. (2004). Biliverdin, a natural product of heme catabolism, induces tolerance to cardiac allografts. FASEB J. 18, 765–767. 10.1096/fj.03-0839fje 14977878

[B164] Yan S. F. Harja E. Andrassy M. Fujita T. Schmidt A. M. (2006). Protein kinase C beta/early growth response-1 pathway: a key player in ischemia, atherosclerosis, and restenosis. J. Am. Coll. Cardiol. 48, A47–A55. 10.1016/j.jacc.2006.05.063 17084284

[B165] Yazicioglu M. N. Goad D. L. Ranganathan A. Whitehurst A. W. Goldsmith E. J. Cobb M. H. (2007). Mutations in ERK2 binding sites affect nuclear entry. J. Biol. Chem. 282, 28759–28767. 10.1074/jbc.M703460200 17656361

[B166] Yoon S. Seger R. (2006). The extracellular signal-regulated kinase: multiple substrates regulate diverse cellular functions. Growth Factors 24, 21–44. 10.1080/02699050500284218 16393692

[B167] Zeng R. Yao Y. Han M. Zhao X. Liu X. C. Wei J. (2008). Biliverdin reductase mediates hypoxia-induced EMT via PI3-kinase and Akt. J. Am. Soc. Nephrol. 19, 380–387. 10.1681/ASN.2006111194 18184861 PMC2396754

[B168] Zhao J. Renner O. Wightman L. Sugden P. H. Stewart L. Miller A. D. (1998). The expression of constitutively active isotypes of protein kinase C to investigate preconditioning. J. Biol. Chem. 273, 23072–23079. 10.1074/jbc.273.36.23072 9722533

[B169] Zhao Y. Bjorbaek C. Moller D. E. (1996). Regulation and interaction of pp90^rsk^ isoforms with mitogen-activated protein kinases. J. Biol. Chem. 271, 29773–29779. 10.1074/jbc.271.47.29773 8939914

